# Multiple impact effects of helium-driven shocks on thin fiber-metal laminates

**DOI:** 10.1038/s41598-023-42861-0

**Published:** 2023-11-09

**Authors:** Anand Pai, Marcos Rodriguez-Millan, Chandrakant R. Kini, B. Satish Shenoy

**Affiliations:** 1 Department of Aeronautical and Automobile Engineering, Manipal Institute of Technology, Manipal Academy of Higher Education, Manipal, Karnataka 576104 India; 2 Department of Mechanical Engineering, University Carlos III of Madrid, Avda. de la Universidad 30, 28911 Leganés, Madrid Spain

**Keywords:** Engineering, Materials science

## Abstract

Fiber Metal Laminates (FMLs) have garnered considerable attention and are increasingly being utilized in the development of protective armors for explosion and ballistic scenarios. While most research has focused on assessing the response of FMLs to single impacts, real battlefield situations often require shielding structures to endure multiple impacts. Thus, this study revolves around the creation of hybrid FMLs designed for shock shielding purposes. The primary focus is on how these laminates withstand repetitive impacts from high-intensity shock waves, aiming to pinpoint the optimal sequence that offers the highest resistance against multiple shock impacts. To establish effective shielding, a multi-layered FML configuration is employed. This configuration incorporates AA6061-T6 facing plates, ballistic-grade synthetic materials like aramid/epoxy ply, and ultra-high molecular weight polyethylene (UHMWPE)/epoxy ply. Additionally, a paperboard/epoxy lamina is introduced to induce functional grading based on layerwise shock impedance mismatches. Shock impact experiments are conducted using a shock tube equipped with helium as the driver gas. Critical shock parameters, including Mach Number, positive impulse, and peak overpressure, are meticulously evaluated. For validation purposes, a numerical model is employed to project the damage profile as a function of radial distance across different laminate sequences. The study unveils that ply deformations are strongly influenced by the arrangement of core layers, particularly the positions of the paperboard and UHMWPE layers within the core structure. To contextualize the findings, the shock impact results obtained from this study are compared with those from prior experiments that employed nitrogen-driven shocks.

## Introduction

In line with the ever-evolving armoury of weapons worldwide, the safety and defense requirements too have become stringent and demanding. The materials used for the shielding structures have undergone a drastic transformation from the early 2000s to the current. In the past four decades, engineers designing the shielding material for protection against impact from high velocity projectiles and shockwaves have resorted to a variety of materials— metallic^1–4^, ceramic^5–7^, fiber-reinforced composites^8–12^, metamaterials like cellular foams^13,14^, and multi-material combinations like metal matrix composites, ceramic matrix composites^15^, cermets, and sandwich structures^16–20^. Sandwich structures are at the forefront of latest research with advancements involving use of novel core materials, modified adhesives, and clamping mechanisms for holding the layers together^21^. This shift in the paradigm of creating hybrid sandwich structures through the fusion of various material types, ushered in the era of Fiber-Metal Laminates (FMLs)^22^.

FMLs combine lightweight, impact-resistant fiber-reinforced polymer plies with high strength and high stiffness (and correspondingly higher density) metallic layers to provide the best qualities of both materials^23,24^. FMLs are a sub-class of sandwich structures in terms of construction. Popular uses for FMLs include structural, impact resistance and shielding applications. In recent years, FMLs like ARALL, CARALL and GLARE have been the focus of in-depth research on mechanical properties, impact resistance to ballistic and blast loads, energy absorption capacities, and thermal resistance^25^. Including ballistic grade fabrics as sandwiched plies along with metallic layers can improve the resistance against the blunt impact by shock waves in addition to protection against ballistic impacts^11,12^. The interlocking weave pattern between fiber yarns (warp and weft) facilitates the distribution of shock energy over a broader area, effectively dissipating the energy across the fabrics. When subjected to shock impact, the high-strength fiber yarns within the woven pattern undergo stretching and deformation, efficiently converting the kinetic energy into mechanical work.

Extreme interest has been shown in the dynamic response of FMLs and multi-layered structures to air-blast loading^26–28^. This has been achieved through an explosive-based direct blast loading, assisted by a ballistic pendulum or the use of appropriate shockwave generating equipment (such as shocktubes) in^29–35^. When the blast wave or shock wave is incident on a target structure, a part of it is transmitted into the structure while another part is reflected. As the transmitted blast wave travels through the structure, it may undergo subsequent reflections at ply interfaces, which depends on the shock impedance mismatch between the adjacent layers. Transmitted high pressure waves lead to positive impulse while reflected pressure wave are responsible for a negative impulse in the corresponding layer. In one of the works, Latourte et al.^9^ examined the failures and deformation mechanisms of sandwiched and monolithic panels with polyvinyl chloride (PVC) foam core subjected to blast loading. Zhou et al.^20^ investigated the air-blast reaction of a FML made of stainless steel faceplate and backplate, sandwich structure containing Divinycell H-series PVC foam core, with suitable adhesive for bonding the layers. They observed local dishing of the faceplate, dome bending at the backplate, core crushing, and delamination at the faceplate/core and backplate/core interfaces. Menkes and Opat^1^ proved that substantial inelastic deformation (Mode 1), rupture at the boundary due to tension (Mode 2), and the supports being sheared (Mode 3) are the three main failure mechanisms in beams subjected to blast loads. Nurick et al.^16^ studied the blast-loading response of sandwich panels made of thin aluminium plates ($\sim$ 0.17 mm thick) and different cores-air-core and hexagonal honeycomb-core made of 5052 aluminium alloy. Detonating a PE4 explosive charge, at a certain stand-off distance from the panels, caused the blast loads. The authors observed that at higher impulses (> 20 Ns), the honeycomb core panels showed lower backplate deformation as compared to air-core panels. Fleck and Deshpande^2^ characterised the three stages of the dynamic reaction of clamped sandwich beams to blast loading: fluid-structure interaction at the impact surface, core crushing or collapse, and bottom plate stretching or bending. Jang et al.^18^ utilized the numerical models for energy absorption, mass, and deflection developed by Xue and Hutchison^3^ to evaluate the performance of sandwich panels against shockwave impact. However, explosion-based air blast studies offer a lot of challenges—high explosives are expensive, require specialized facilities for controlled explosions, demand strict protocols for safety, lack of reproduceability between successive charge detonation, and environmental considerations. Due to the limitations of charge detonation based blast-impact studies, compressed driver gas-based shocktubes were preferred due to their relative safety, ability to induce dynamic loading, precise and accurate diagnostics through high sensitivity pressure transducers, but the specimen size was limited by the shocktube diameter. Additionally, extensive numerical studies have been carried out to comprehend the impact of blast waves on structures, predominantly involving mild steel and armor steel plates^36–38^. The numerical models were able to capture the transient deformation history of the structures, utilizing the physics of fluid-structure interaction, and multi-material Eulerian approaches^39,40^. In some of the numerical analyses of sandwich structures, the material models employed comprise the Cowper–Symonds for strain-rate sensitivity^41,42^, Johnson–Cook for the flow stress^43^. Other damage models have been employed in Refs.^44–46^ for predicting the response of fiber-reinforced composites and laminates.

Pai et al.^47–50^ have extensively worked on FMLs for mechanical, and air-blast resistance characterization. In these works, the influence of the grading of the plies on the response of the FMLs have been investigated in detail. Apart from AA6061-T6 outer plates, the core layers were made of ballistic grade fabrics, and a different material class in paperboard was inserted as a mode for inducing the shock impedance mismatch. Paperboard has been known to display a naturally occurring out-of-plane auxetic behaviour, while its in-plane positive Poisson’s ratio render them partially auxetic^51–53^. Paperboards also have a relatively low value of shock impedance^54^. When paperboard is placed adjacent to UHMWPE or metallic plates, the final transmitted shock pressures could be moderated offering an overall shock attenuation to the incident blast waves. However, shock impact analysis for helium-driven, higher-intensity shock waves was not covered in these works.

The objective of the current work is to experimentally investigate the response of various configurations of thin FMLs subjected to multiple shock impacts using helium-driver gas inside a shocktube with validation through numerical approach. Five configurations of FMLs with AA6061-T6 outer layers, and core layers consisting of Aramid-epoxy, UHMWPE-epoxy, and paperboard-epoxy are used for shock impact studies. For validation, using the boundary conditions existing for the specimen inside the shock tube, a numerical model is created to predict the deformation profile of the laminates upon shock impact. The corresponding material properties of the plies are computed using the representative volume element (RVE) approach employing the MATERIAL DESIGNER module. The stackups are built on the ANSYS COMPOSITE PREP/POST (ACP) to obtain the laminate properties. The loads applied in the numerical model are derived from the outcomes of the experiments. The numerical model predicts the deformation profiles of the FMLs subjected to shock wave impact, combining the approaches of RVE, Stackup construction, and helium-driven shock parameters from the shock tube experiments, which serves as the novelty in this work. The dimensionless deformation for the laminate computed by the numerical model is compared with that of the experimental results. Cone beam computed tomography (CBCT) is used to study the shock-impacted specimens of the sequences, and identify the failure modes and mechanisms of the FMLs. The relationship between the ordering of the layers within the core based on shock impedance matching, and the deformation profiles, failure modes, and shock shielding capabilities is studied. FMLs displaying least deformation offer the best protection against shockwave impact. Thus, this study enhances the understanding of mechanical response of FMLs to repeated shockwave impacts, and the numerical model can be used in other material systems. The inclusion of partially auxetic layers within the core and the location of the ply also influences the response of the FMLs. The research enhances knowledge on FML behavior under dynamic loads and provides valuable insights for designing impact-resistant materials. The findings hold significance for aerospace, defense, and automotive industries. However, the numerical model may fail to predict the FML responses at close proximity to the blast epicentre, since the shockwave propagation is three-dimensional.

## Materials and methods

**Figure 1:**
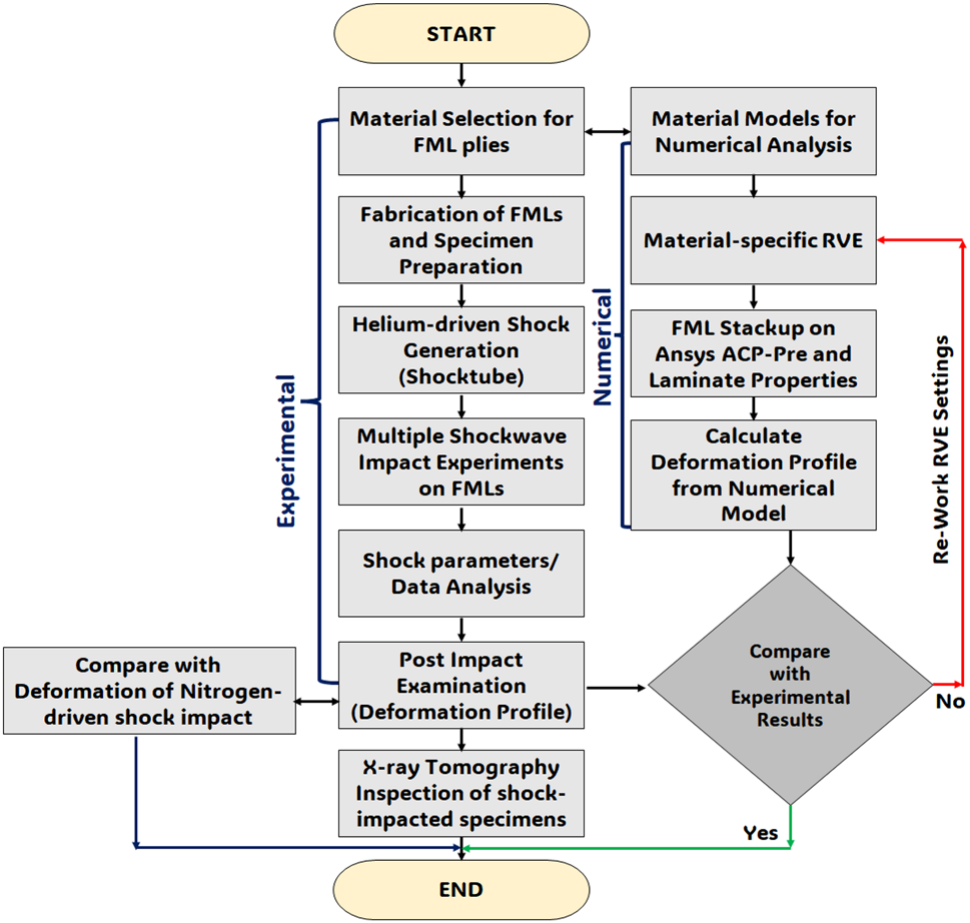
Algorithm for the validation of the experimental analysis through the numerical model.

The experimental and numerical approaches used in the multiple shockwave impact effect studies on the FMLs can be inferred from Fig. 1. The experimental approach comprises the material selection for the plies, FML sequence design, fabrication and specimen preparation, shocktube experiments with helium-driven shocks, and post-impact analysis with X-ray tomography. The numerical approach consists of selection of appropriate material models for the various plies, the development of material-specific unit cell by Representative Volume Element (RVE) method, creation of FML stackup on ANSYS ACP-Pre module, extraction of laminate properties, substitution in the numerical model and computation of the deformation profile. The validation involved comparing the deformation profiles from the experimental and numerical approaches, and alterations in the RVE settings for establishing the accuracy of the deformation profile predicted by the numerical model.

### Materials

The laminates were constructed using a multi-layered sandwich configuration, in which metallic plates sandwiched the core layers made of ballistic grade fabrics of aramid, UHMWPE, and a separate material class paperboard-epoxy ply for the purpose of shock tuning. Metallic plates play the role of high strength, high shock impedance layers, which attenuate the transmitted shock pressures propagating into successive layers of lower shock impedance^17,37^. Subsequent to the metallic plates, ballistic grade aramid fabric was strategically positioned to provide impact resistance against blast debris and shrapnel that might breach the metallic plates. Likewise, ballistic grade UHMWPE would serve a similar role on the rear side of the core configuration. The metallic plates were made of AA6061-T6 sheets, to serve as the faceplate and backplate (distal plate), core layers comprising UHMWPE, aramid, and paperboard plies, and epoxy binder. The epoxy resin used in the work was $CT/E - 556$ along with a polyamine hardener *CT*/*AH*?951 (ratio of the resin to hardener was 100:10 by weight). The resin and hardener were supplied by M/s Composites Tomorrow, India. The arrangements of the core layers were altered based on functional grading taking into account the shock impedance mismatch as a metric. Overall, five sequences were taken up for the shock impact investigation.

**Figure 2:**
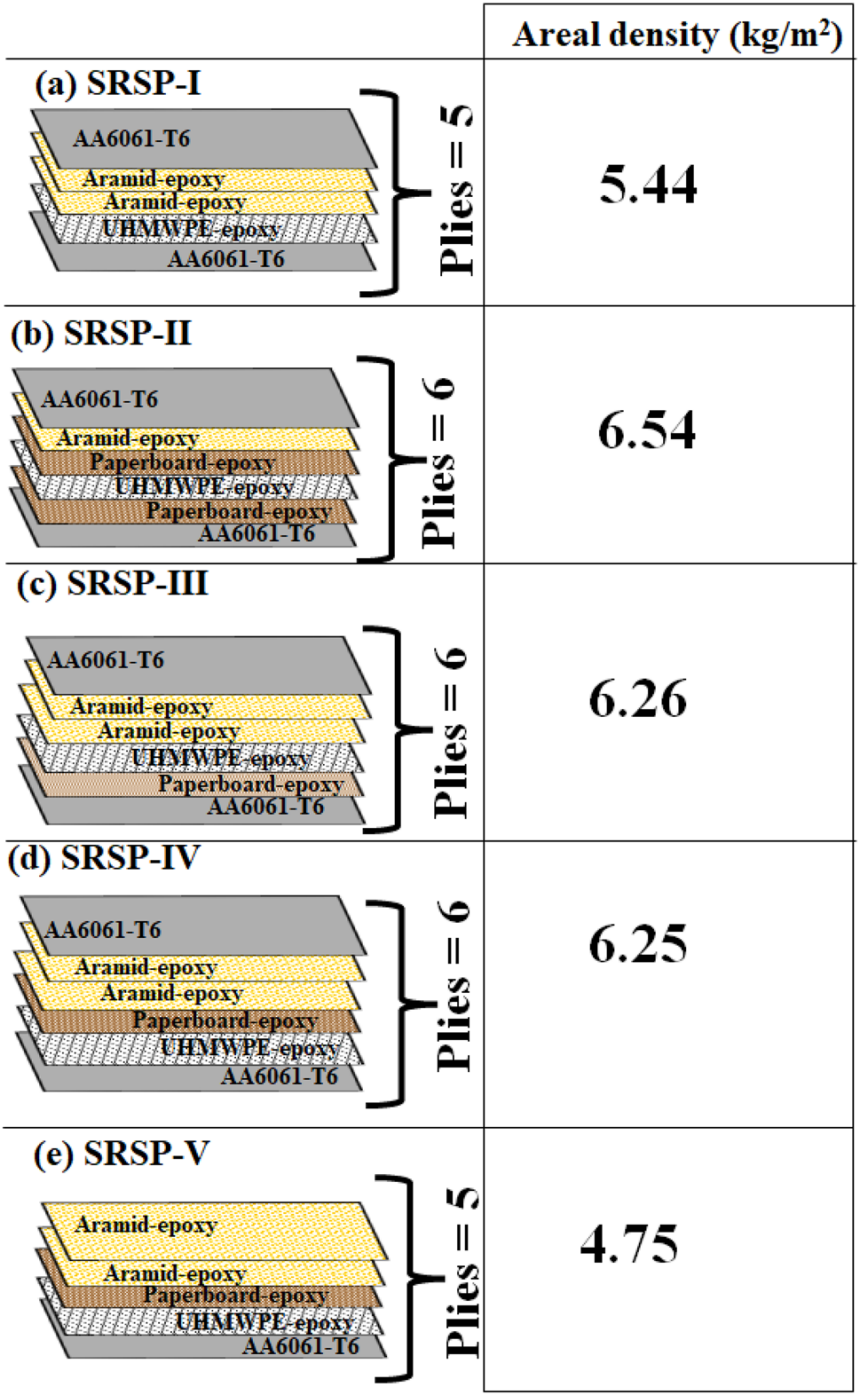
Representation of different stacking sequences with dimensions (**a**) SRSP-I (**b**) SRSP-II (**c**) SRSP-III (**d**) SRSP-IV (**e**) SRSP-V.

AA6061-T6 plates (0.7 mm thick) were procured from Hi-Tech Sales Corporation in Mangalore, India. The AA6061-T6 plates were subjected to mechanical abrasion in order to give them a rough finish that would facilitate proper wetting by the resin. The aramid plies (0.3 mm and 0.6 mm thick), UHMWPE plies (0.25 mm thick and 0.5 mm thick), and epoxy binder were sourced from Composites Tomorrow in Gujarat. In addition, paperboard sheet (0.25 mm thick) was supplied by Vijay Papers, India. Figure 2 demonstrates the layering order for the five sequences used in the study, also highlighting the areal densities of the sequences. The different sequences were fabricated by hand-layup followed by compaction on a compression moulding machine. Each laminate was then cut by water jet machining to create round specimens of 100 mm diameter for shock tube experiments. Post-machining, the specimens were inspected for damages related to machining like delamination or debonding, by scanning the cut specimens using CBCT. The CBCT scanned images were investigated along the ply edges and ply interfaces for defects. However, none of the specimens/ sequences exhibited any signs of delamination or debonding, facilitating further studies on the shock tube.

### Experimental methodology

**Figure 3:**
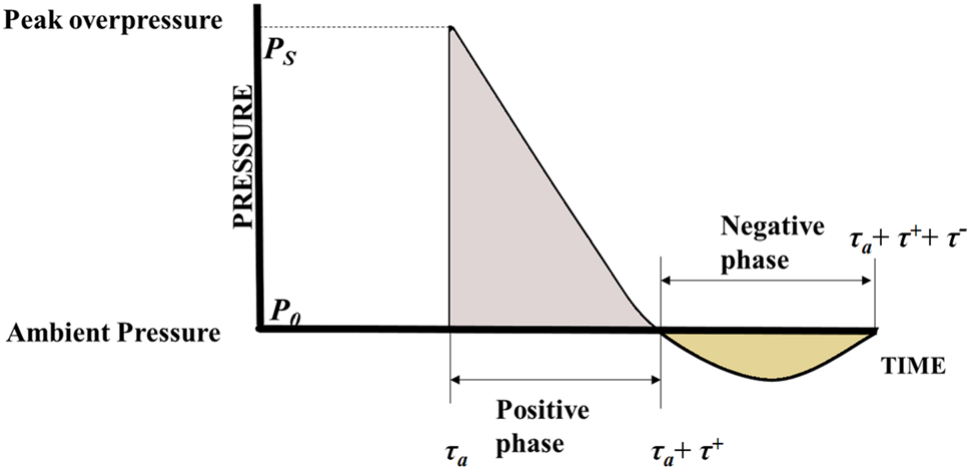
Friedlander profile of blast waves^26^.

According to the Friedlander curve^26^, the pressure at the contact face increases to a peak overpressure value before declining as seen in Fig. 3. Equations (1), (2) and (3) show the time-rate of momentum change, the blast impulses in a typical blast wave. The change in momentum ${\Delta }(mu)$ depends on the wave pressure ‘*P*’, the flow area ‘*A*’ over a time period ‘*dt*’. The positive impulse $I^+$ acts during the time instant (t$_a$) to (t$_a$+t$^+$), with *P*(*t*) being the pressure at any time instant ‘*t*’, P$_0$ being the ambient atmospheric pressure. The negative impulse $I^-$ occurs during the time instant (t$_a$+t$^+$) to (t$_a$+t$^+$+t$^-$). The overpressure, positive and negative impulses, and the intensity of the blast wave are interdependent, with the positive impulse primarily causing the high-pressure loading on the structure. The integration in Eqs. (1–3) is achieved by means of numerical integration through the Trapezoidal rule.

1 $$\begin{aligned} \Delta (mu)= & {} \int _{}^{} PA \,d\tau \ \end{aligned}$$

2 $$\begin{aligned} I^+= & {} A\int _{\tau _a}^{\tau _a+\tau ^+} (P(\tau )-P_0) \,d\tau \ \end{aligned}$$

3 $$\begin{aligned} I^-= & {} A\int _{\tau _a+\tau ^+}^{\tau _a+\tau ^++\tau ^-} (P_0-P(\tau )) \,d\tau \ \end{aligned}$$

**Figure 4:**
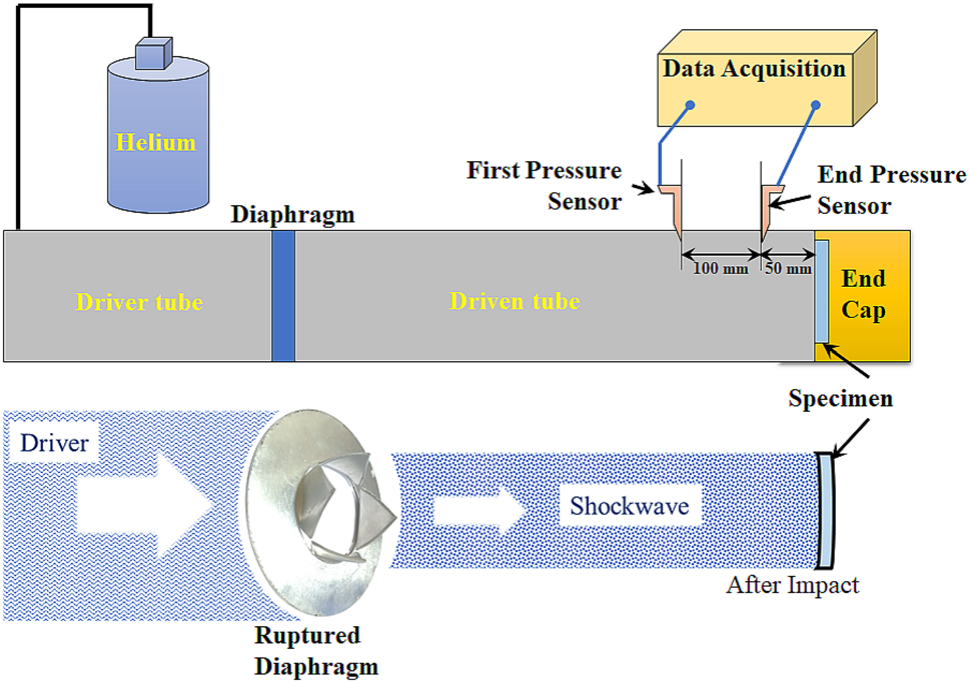
Shocktube experimental setup with helium driver gas^48^.

A high-pressure shock tube was used for the shock impact experiments. In our previous work^48^, the specifications and the experimental settings have been described. Helium gas was chosen in the current study as the driver. Being an inert gas with an adiabatic index of 1.67 and the second highest mobility (next to hydrogen gas), it is capable of generating higher Mach numbers on the same shock tube with aluminium diaphragms ($\sim$ 2 mm thick)^34,35^. The serration, thickness, and material of the diaphragm influence the shock velocity, decay time, and $P_5$ pressure^33^. An end cap recess on the rear flange was used to hold the laminate specimens (100 mm-diameter). The specimen was located 50 mm behind the End pressure (EP) transducer as seen in Fig. 4. The First Pressure (FP) Sensor was fitted 100 mm ahead of the End pressure sensor towards the driver side. As the driver section pressure was increased, on regulating the volume of the driver gas (*He*), beyond a certain pressure, the scribed diaphragm broke, sending shockwaves toward the target plate as demonstrated in Fig. 4. Two side-on pressure transducers (Make: PCB113B22, Measurement range: $\sim$ 34.5 MPa, Sensitivity (± 10%)1.0 mV/kPa) that were spaced apart by an axial distance of 100 mm captured the pressure-time data of the shockwaves impinging on the specimen.

The data acquisition device comprising a three channel oscilloscope (Make: HANTEK) and signal conditioner (Make: PCB Piezotronics) collected the pressure-time information for the shockwave. Each specimen of a particular sequence was first subjected to a single shock impact, after which measurements of the deformation profile were taken, and the specimen was then exposed to a second shock impact in the shocktube. Repeated measurements of the deformation profile were taken. The shock Mach number can be obtained using Eqs. (4), (5) and (6). $V_s$ refers to the shock speed which is inversely proportional to the time lapse ‘$\Delta t$’. Sound velocity in air ‘*a*’ depends on the adiabatic index of air ‘$\gamma$’, ambient temperature ‘*T*’ (= 27–28 °C), and the gas constant ‘*R*’. Shock Mach Number ‘*M*’ is then expressed as a ratio of the shock speed to the acoustic speed. During each shock tube experiment, the data acquisition device yielded the pressure time data which was used to construct the pressure-time plots. The pressure-time plots helped obtain the key metrics: Shock speed $V_s$, the time lapse $\Delta t$ between the respective pressure rise for the FP and EP sensors, the $P_2$ level pressure (initial pressure due to the shockwave as recorded by the FP and EP sensors), $P_5$ level pressure (maximum pressure on the *P*–*t* plot) and the decay time ‘$t_d$’ (which is defined as the time gap between the instant of pressure rise to the time when the pressure drops down to the initial level). $P_4$ pressure refers to the maximum driver pressure at the instant of diaphragm rupture obtained from the driver pressure guage (diaphragm separating the driver and driven sections of the shock tube). $P_1$ refers to the initial pressure inside the driver tube. The shock characteristics for each shockwave impact on the test specimen by the helium-driven shock were computed using SCILAB codes.

**Figure 5:**
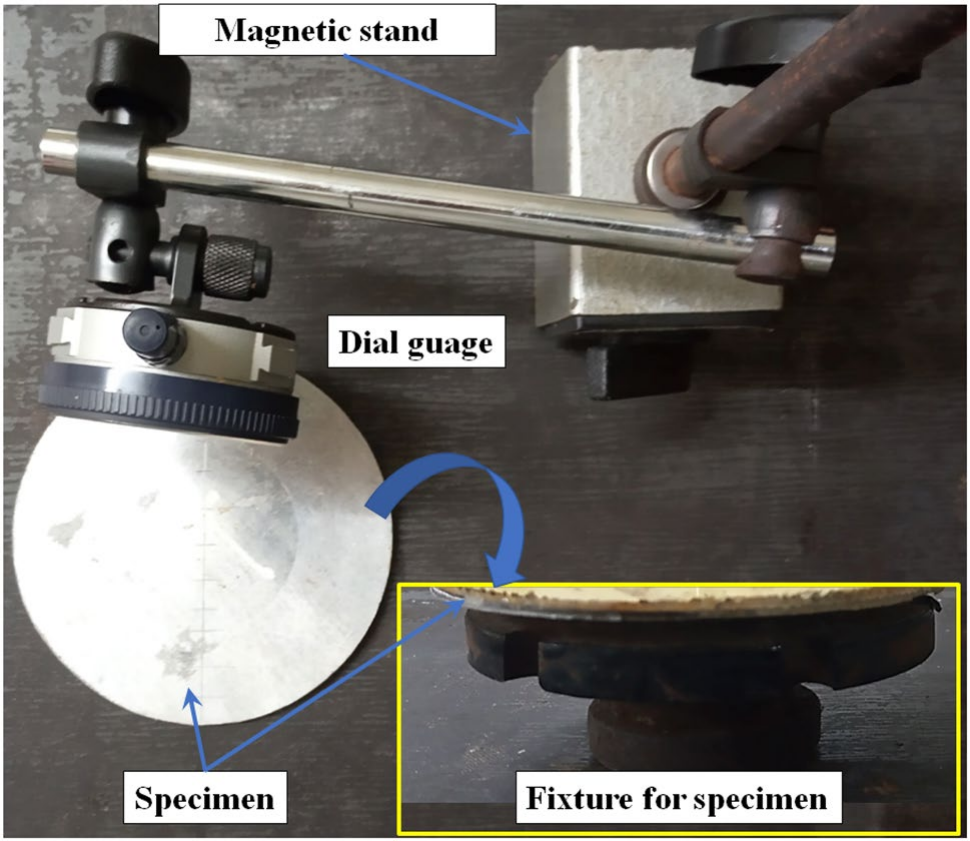
Photograph of Dial guage and fixture setup to measure the deformations.

4 $$\begin{aligned} {V_s}= & {} \frac{0.1}{\Delta t} \end{aligned}$$

5 $$\begin{aligned} {a}= & {} \sqrt{\gamma R T} \end{aligned}$$

6 $$\begin{aligned} {M}= & {} \frac{V_s}{a} \end{aligned}$$

To measure the faceplate and backplate deformations, the specimens were placed on suitable fixtures mounted on a surface plate and a dial guage was used to measure the deformation values by moving the tip across the diameter of the specimen as shown in Fig. 5. The diametric measurements were repeated and average deformations for the faceplate, and the backplate were taken for analysis.

### Numerical modeling

To develop the numerical model, the individual behaviour of the constitutive plies in the five sequences had to be taken up. The elastic behaviour of AA6061-T6 was considered as isotropic and homogeneous while the other plies displayed orthotropic elasticity. To account for the plastic behaviour of aluminium alloy, a von Mises yield criterion was taken up^43^. Regarding the modeling of aramid-epoxy, UHMWPE, and paperboard layers, they have been modeled as elastic until failure. As no penetration or perforation damage was observed, the damage model, strength model, and failure models of the plies were not taken into account^10–12^.

**Figure 6:**
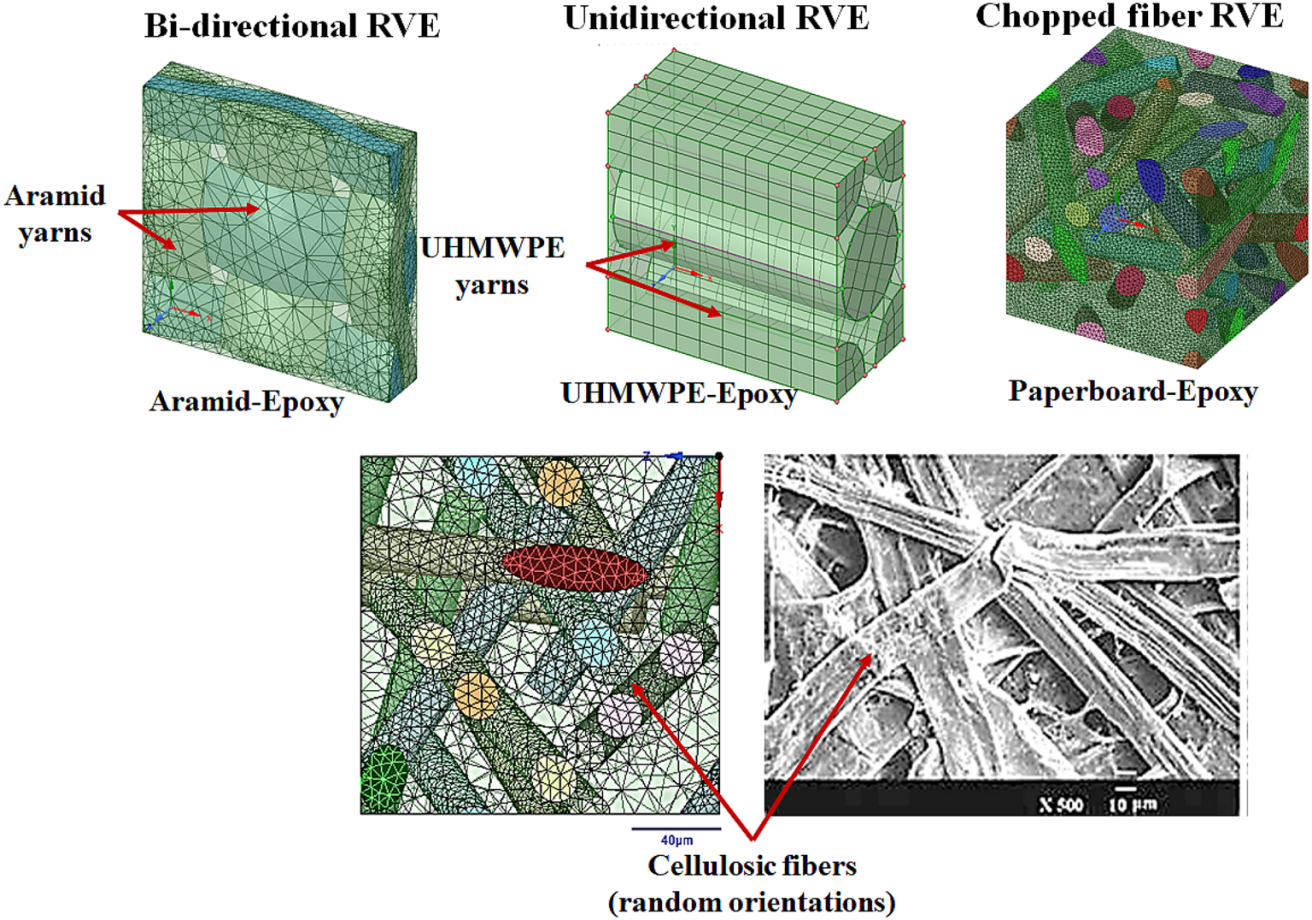
RVE unit cells of aramid-epoxy, UHMWPE-epoxy and paperboard-epoxy layers.

**Table 1:** Materials properties of the FML sequences from RVE on MATERIAL DESIGNER.

Properties	AA6061-T6	Aramid-epoxy	UHMWPE-epoxy	Paperboard-epoxy
Density ($\text {kg/m}^3$)	2760	1380	1210	1250
$E_{1}$ (GPa)	69	13.7	3.23	3.95
$E_{2}$ (GPa)	69	13.7	3.24	3.86
$E_{3}$ (GPa)	69	4.96	3.01	4.06
$\nu _{12}$ (GPa)	0.33	0.52	0.37	0.304
$\nu _{23}$ (GPa)	0.33	0.263	0.41	0.197
$\nu _{31}$ (GPa)	0.33	0.263	0.37	0.226
$G_{12}$ (GPa)	25.9	4.84	1.1	1.51
$G_{23}$ (GPa)	25.9	2.91	1.07	1.50
$G_{31}$ (GPa)	25.9	2.91	1.09	1.57
Thickness (mm)	0.8	0.45	0.32	0.4

**Figure 7:**
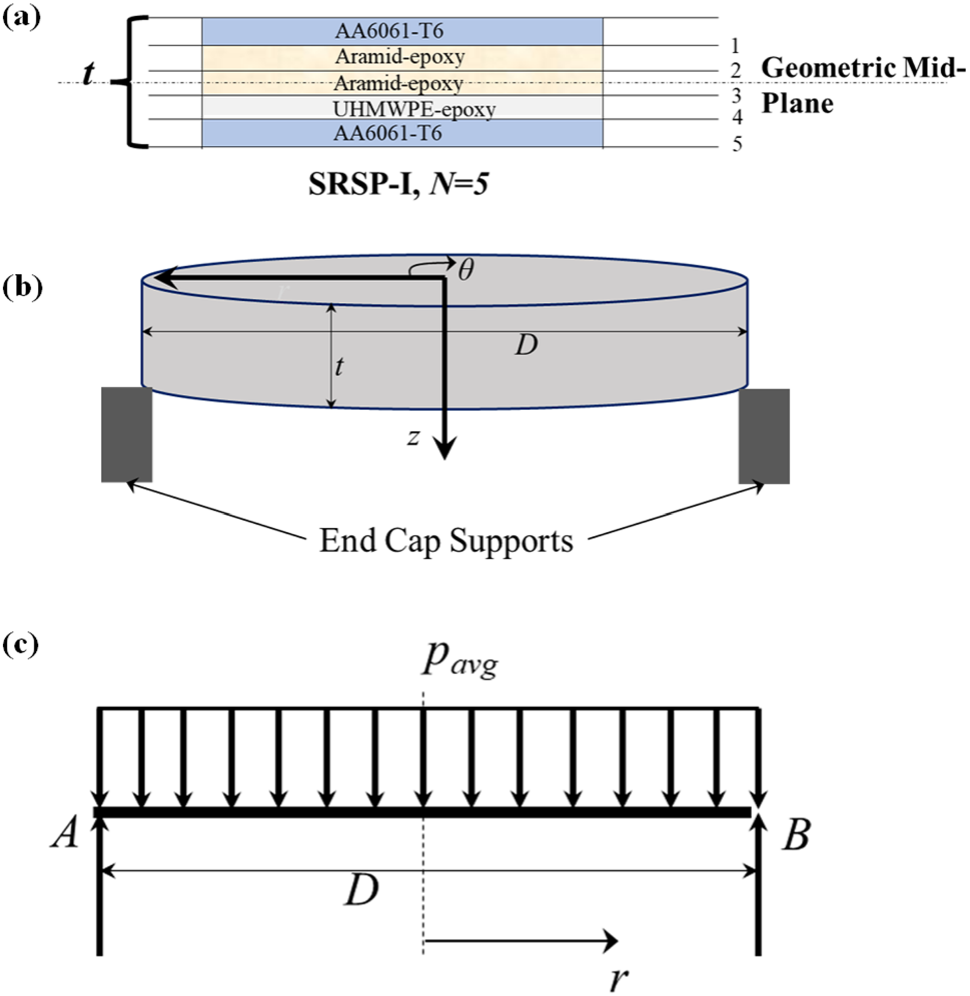
(**a**) Laminate Stackup for SRSP-I (**b**) Specimen subjected to shock impulse (**c**) Beam loaded with constant pressure ‘$p_{avg}$’ as summarized in Tables 3 and 4.

**Figure 8:**
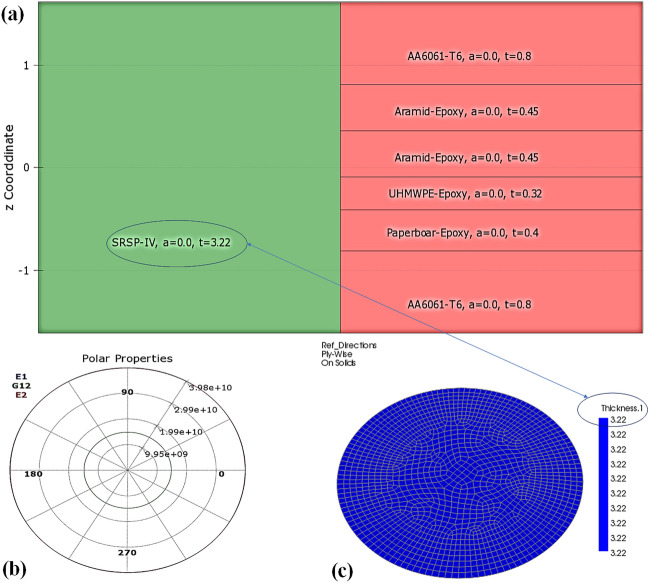
Numerical model of stackup SRSP-IV developed on ANSYS ACP-Pre for determining the laminate engineering constants based on CLT (**a**) Stackup of SRSP-IV (**b**) Computed engineering properties of SRSP-IV (**c**) Meshed model.

Using the RVE approach on the MATERIAL DESIGNER module of ANSYS R20.0,the orthotropic properties of the plies were computed with a similar approach to one of our recent works^48^. An isotropic RVE was used for the AA6061-T6 layer, while a bidirectional (BD) RVE was chosen for the Aramid-epoxy ply due to its bidirectional fabric structure. For the UHMWPE-epoxy ply, a unidirectional (UD) RVE was employed, matching its unidirectional fabric orientation. Regarding the paperboard-epoxy ply, a chopped fiber RVE was selected in MATERIAL DESIGNER, where the fiber orientation was random. This randomness can be attributed to the paperboard’s composition, consisting of cellulosic fiber networks with fiber lengths ranging from 0.5 to 2 mm, forming a non-woven network held together by hydrogen bonding at various points^51^. The RVE unit cells for the materials are shown in Fig. 6. The mechanical properties along the three principle axes for each of the plies were calculated from the MATERIAL DESIGNER and tabulated in Table 1. The numerical model was based on the classical laminate theory^40^ with following assumptions:

- The individual plies of the laminates apart from AA6061-T6 behaved orthotropically (modelled elastic until failure), with the primary material axes positioned along global x and y axes, respectively.
- Each ply has a constant thickness “$t_p$”, ($<<$ diameter of the circular specimens ‘*D*’).
- The interfaces of the plies are flawlessly bonded, and any porosity within the plies is neglected.
- Since circular laminate plates are used as test specimens for shocks, (Fig. 7a), by applying the symmetry about the centre, the pressure loading on the faceplate might be depicted as an example of a simply supported beam with evenly distributed loading. The circular plate can be obtained when the beam in Fig. 7c is rotated through the angle ‘$\theta$’.
- Driver gas pressure (Helium) acts during the positive impulse phase for the single shock impact^30^, over a decay time of 7–7.6 ms. Hence, the average pressure $p_{avg}$ equal to the positive impulse divided by the decay time. The beam is subjected to this average pressure, as seen in Fig. 7c. In numerical models, the distributed loaded beam strategy has been employed in the past^3^. The values of ‘p$_{avg}$’ are summarized in Tables 3 and 4.

In a *N*-ply laminate (for instance, the SRSP-I sequence in Fig. 7b), the longitudinal stresses are connected to the applied bending moment $M_b$ by Eq. (7). The moment-curvature relationship from materials science^40^ shown in Eq. (8) when combined with Eq. (7) gives Eq. (9), where $E_{f}$ is the equivalent flexural modulus for the laminate. $I_{yy}$ the polar moment of inertia around the geometric mid-plane is given by Eq. (12). The stackups of each sequence were created using the ANSYS ACP-Pre module, $E_f$ for the different sequences was subsequently determined. The numerical model of the stackup for one of the sequences, SRSP-IV is shown in Fig. 8. Figure 8a shows the layers in the stackup, while Fig. 8b shows the engineering constants while Fig. 8c shows the numerical model. After the grid independence check, a mesh size of 0.05 mm was chosen for the stackups resulting in 1569 elements. For each sequence, the effective laminate flexural modulus ($E_f$), laminate stiffnesses ($E_x$ and $E_y$), and out-of-plane shear correction ($K_{44}$) and ($K_{55}$) coefficients were determined. The transverse deflection is given by Eq. (11) (along the z-direction), and it is based on the deflection of beams with hinged end-supports and evenly distributed stress. The computed deformation responses with those of shock impact were compared for each sequence. The ratio of maximum transverse deflection after the first shock impact, ‘$w_{max,1}$’ to laminate thickness ‘*t*’ was used for each sequence as a key metric for comparison (first shockwave impact). The maximum transverse deflection after the second shock impact ‘$w_ {max,2}$’ was determined by adding the transverse deformation ‘$w_{max,1}$’ to that calculated from the second hit using Eq. (14). SCILAB codes were employed for the computation in Eqs. (10) to (14) and for generating the different plots. The laminate properties of the different sequences has been shown in Table 2.

7 $$\begin{aligned} M_b= & {} \sum _{j=1}^{N} \frac{b\left( E_{x}\right) _{j}\left( {z^3_{j}}-{z^3_{j-1}}\right) }{3\kappa } \end{aligned}$$

8 $$\begin{aligned} M_b= & {} \frac{E_fI_{yy}}{\kappa } \end{aligned}$$

9 $$\begin{aligned} E_f= & {} \sum _{j=1}^{N} \frac{b\left( E_{x}\right) _{j}\left( {z^3_{j}}-{z^3_{j-1}}\right) }{3I_{yy}} \end{aligned}$$

10 $$\begin{aligned} w_1= & {} \frac{24p_{avg,1}D^2r^2-16p_{avg,1}r^4-5p_{avg,1}D^4}{384E_{f}I_{yy}} \end{aligned}$$

11 $$\begin{aligned} w_{max,1}= & {} \frac{-5p_{avg,1}D^4}{384E_{f}I_{yy}} \end{aligned}$$

12 $$\begin{aligned} I_{yy}= & {} \frac{bh^3}{12} \end{aligned}$$

13 $$\begin{aligned} w_2= & {} \frac{24p_{avg,2}D^2r^2-16p_{avg,2}r^4-5p_{avg,2}D^4}{384E_{f}I_{yy}}+w_1 \end{aligned}$$

14 $$\begin{aligned} w_{max,2}= & {} \frac{-5p_{avg,2}D^4}{384E_{f}I_{yy}}+w_{max,1} \end{aligned}$$

**Table 2:** Laminate properties obtained from the numerical model on ANSYS ACP-Pre for different sequences.

Sequence	$E_{f}$ (GPa)	$E_{x}$ (GPa)	$E_{y}$ (GPa)	$K_{44}$	$K_{55}$	$I_{yy}$ ($\text {mm}^4$)
SRSP-I	24.34	44.15	44.12	0.154	0.156	1302
SRSP-II	23.40	38.37	38.33	0.129	0.133	3888
SRSP-III	23.25	39.81	39.20	0.149	0.152	3573
SRSP-IV	23.26	39.80	39.18	0.148	0.151	3573
SRSP-V	15.02	29.17	29.12	0.182	0.187	1829

### Cone beam computed tomography

**Figure 9:**
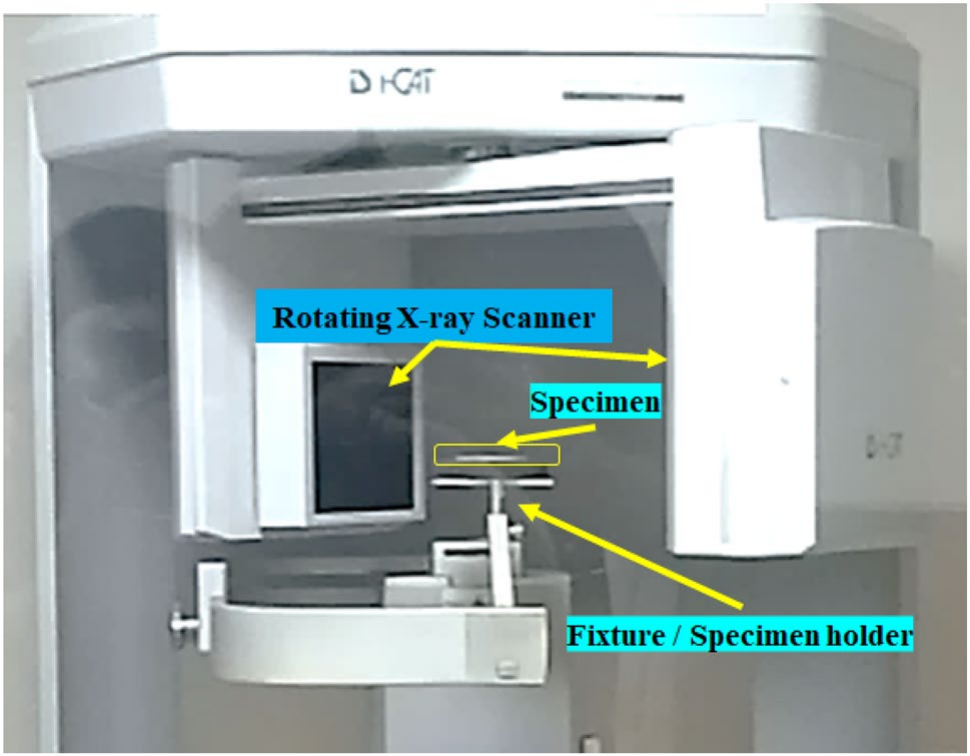
CBCT Scanner with specimen mounted on the holder^48^.

Using the i-CAT CBCT scanner, the specimens that had been subjected to the twin shock hits were examined independently. In the cone-beam technique, the X-ray head rotates continuously in a 360°scan, focussing on the specimen placed on a suitable fixture. Figure 9 shows the specimen placed for CBCT scanning. The feed from the X-ray head was fed to INVIVO DENTAL VIEWER software for reconstructing images in the axial, sagittal, and coronal planes? Three mutually orthogonal planes. The as-machined sequences were subjected to CBCT scanning for inspecting any defects in the specimens after water jet machining. The CBCT scanning for the sequences was repeated after subjecting the specimens to helium-driven shock impact.

**Figure 10:**
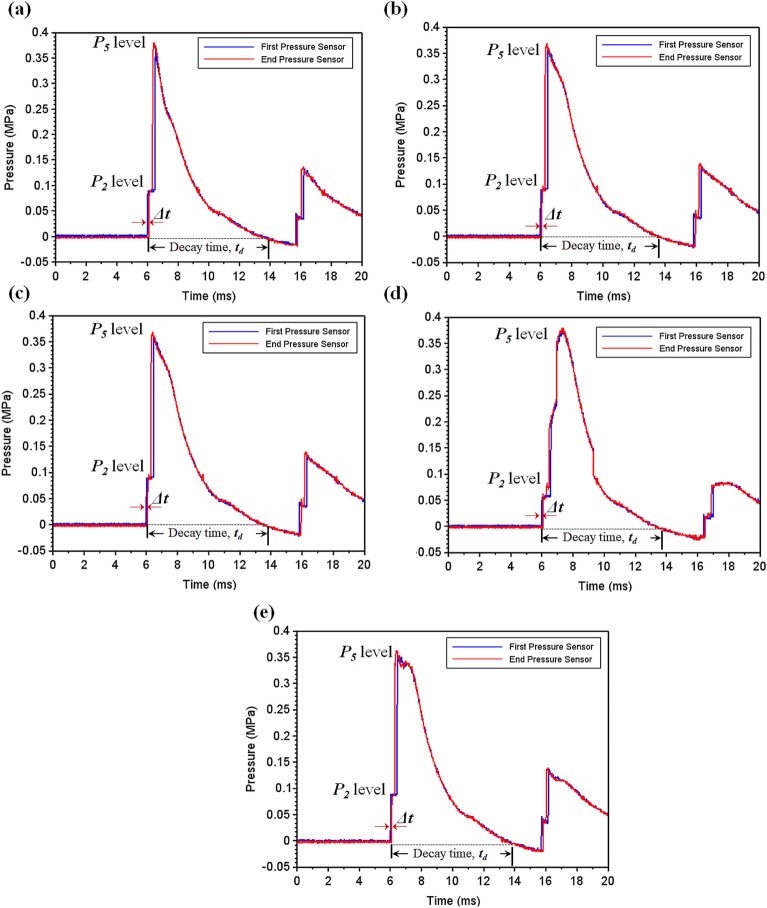
Shock wave interaction pressure-time plot for a single shock hit (**a**) SRSP-I (**b**) SRSP-II (**c**) SRSP-III (**d**) SRSP-IV (**e**) SRSP-V.

**Figure 11:**
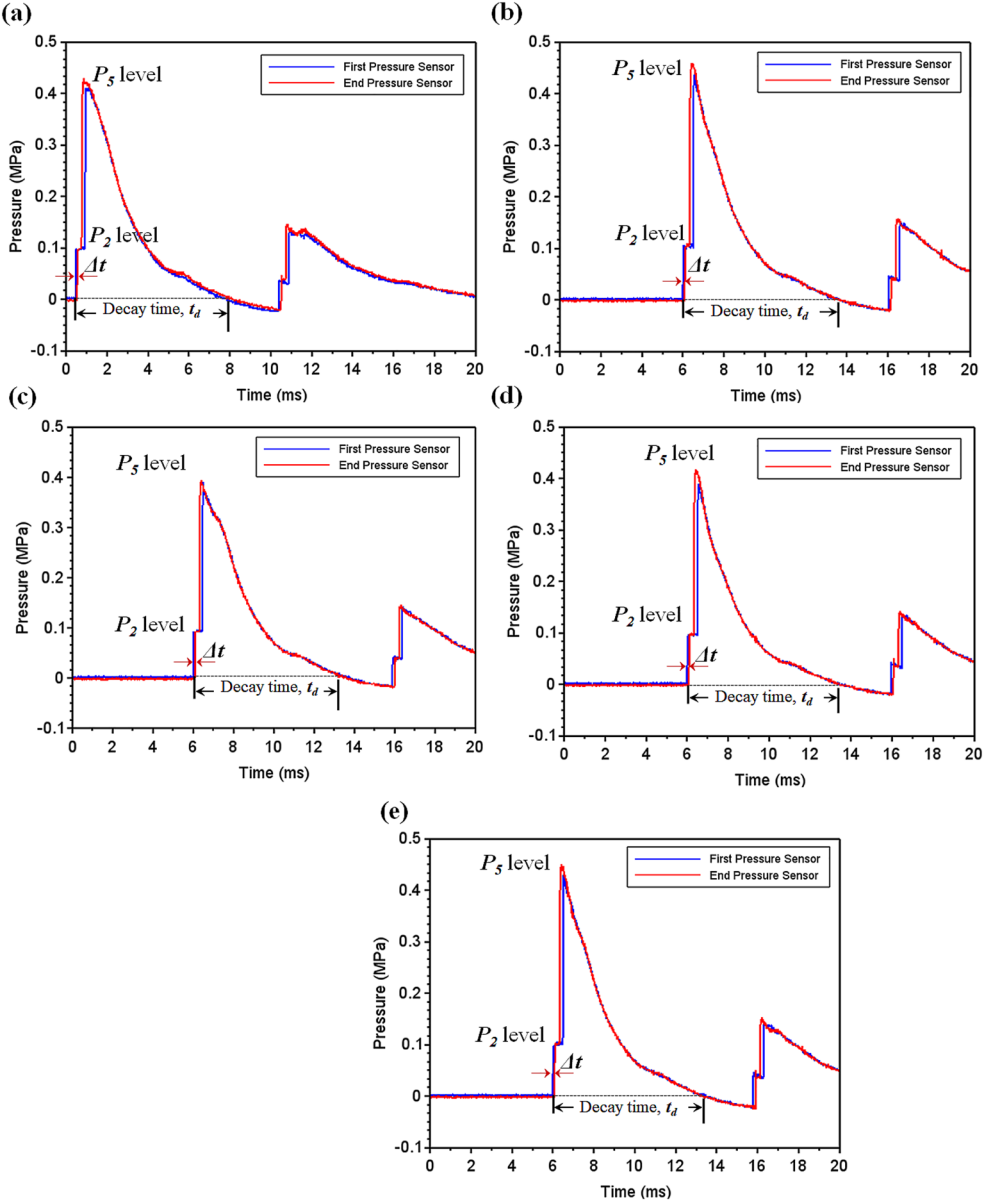
Shock wave interaction pressure-time for second shock hit (**a**) SRSP-I (**b**) SRSP-II (**c**) SRSP-III (**d**) SRSP-IV (**e**) SRSP-V.

**Figure 12:**
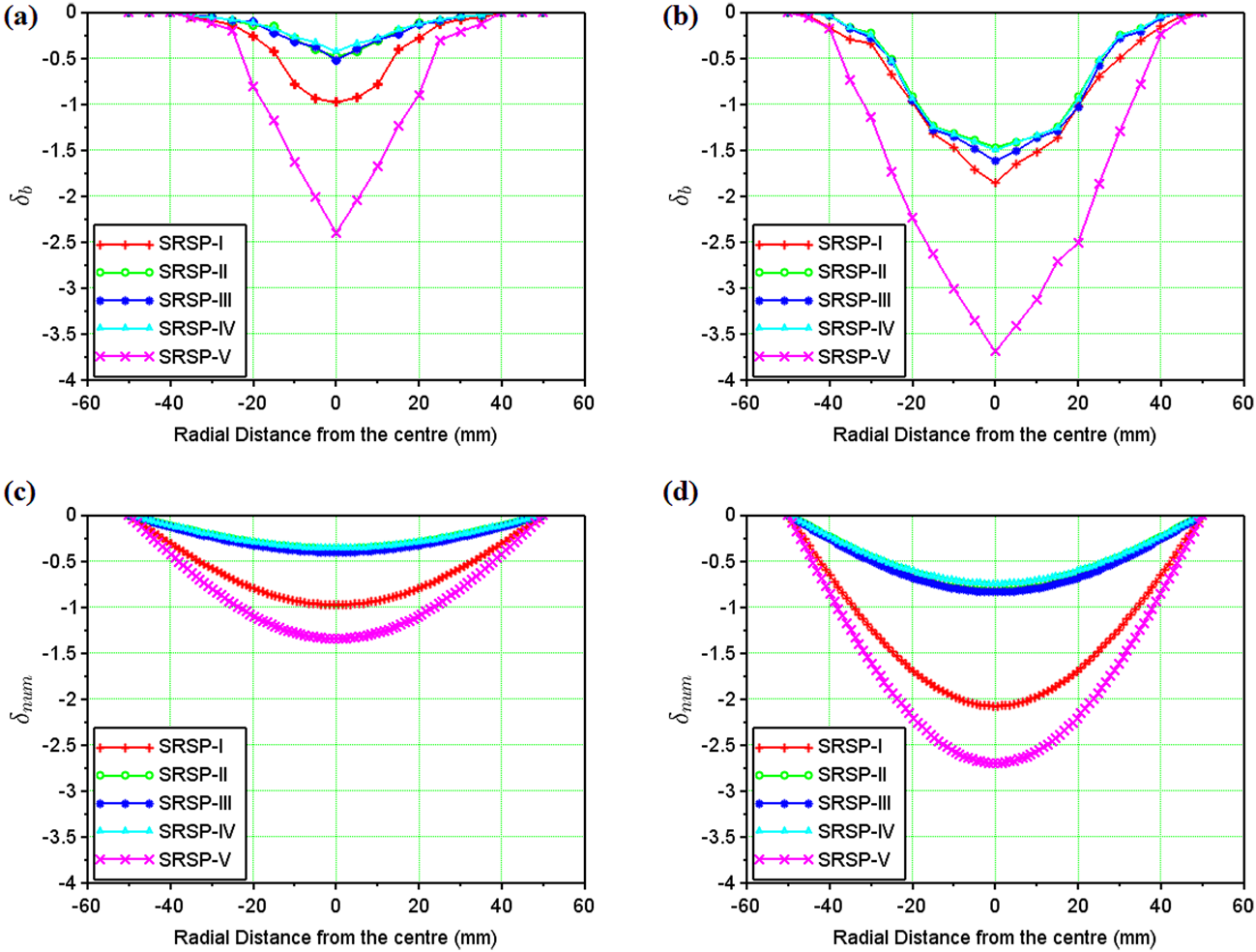
Backplate deformation (mm): (**a**) Experimental (Single Hit); (**b**) Experimental (Second Hit); (**c**) Numerical (Single Hit); (**d**) Numerical (Second Hit).

**Figure 13:**
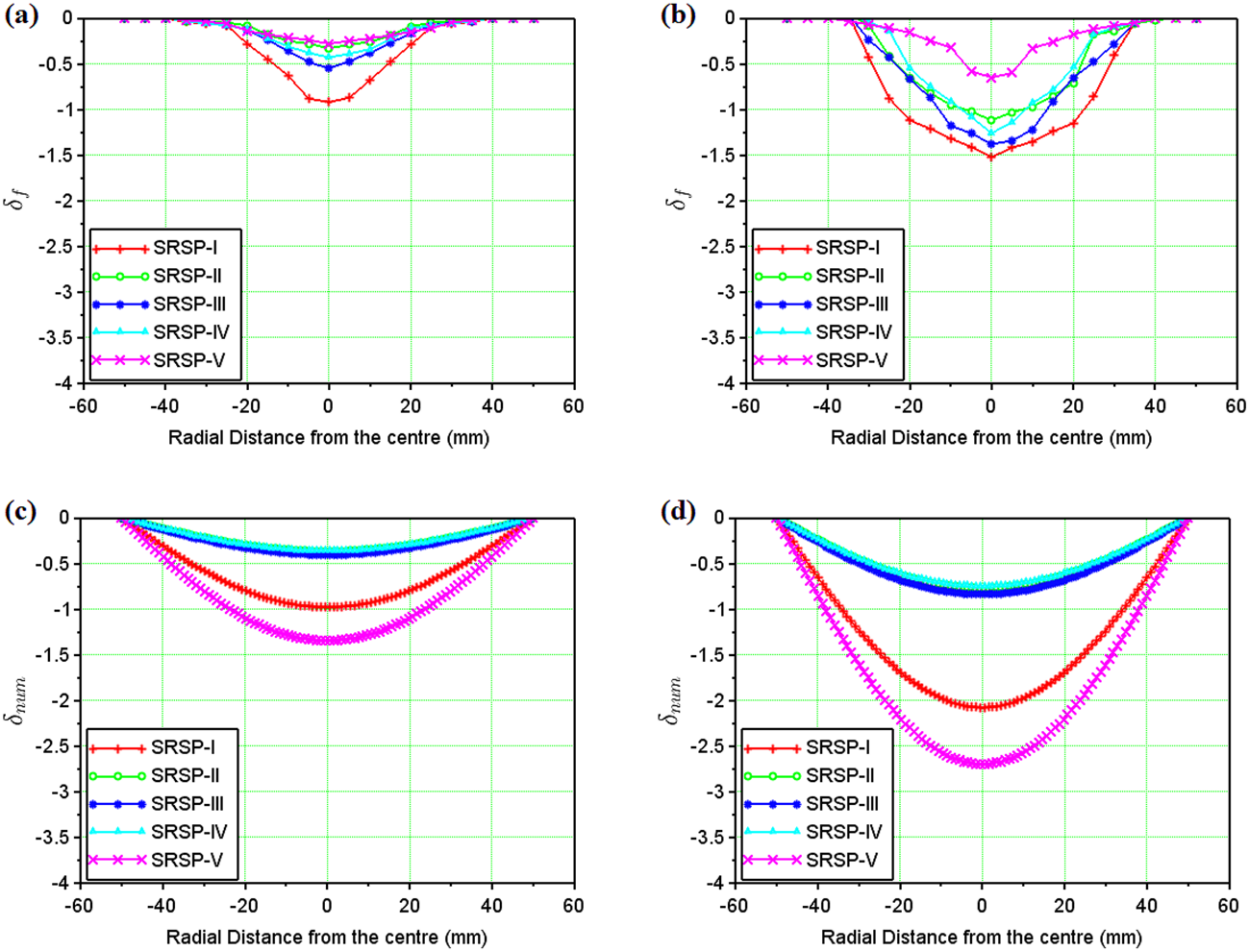
Faceplate deformation (mm): (**a**) Experimental (Single Hit); (**b**) Experimental (Second Hit); (**c**) Numerical (Single Hit); (**d**) Numerical (Second Hit).

## Results and discussion

### Experimental results of shock impact on the FMLs

As the diaphragm separating the driver and driven sections of the shocktube ruptures, a compression shockwave travels through the driven side and at the same time, a rarefaction wave through the driver side. The pressure on the driven side of the shocktube increases to P$_2$ (pressure behind the shockfront) at $\sim$ 6 ms and subsequently to the peak pressure level of P$_5$ (pressure behind the reflected shock) at $\sim$ 7 ms^48^. Afterwards, there is a gradual decrease to the initial pressure value, over a decay time ‘t$_d$ (from $\sim$ 7 ms to $\sim$ 15 ms), with further interactions between the rarefaction waves and shockwaves. The shock wave propagation in the driven tube triggers the signals from the First Pressure and End Pressure sensors. These signals are recorded by the data acquisition unit as voltage-time data. Due to the distance of 100 mm between the sensors, a time lapse $\Delta t$ exists between the pressure-time data which is evident in Figs. 10 and 11, the pressure-time plots recorded during the first and subsequent second shock impact respectively. Table 3 gives the specifics of the single shock impact on each of the five sequences. During the single shock impact studies, the Mach Numbers were found to be in the range of 2.89–2.95, with a decay time of 7.81–7.86 ms. Table 4 shows the double shock impact details and shock parameters. Mach numbers were found to vary from 3.12 to 3.34, the shock velocities were found to vary between 1080 and 1156 m/s, while the decay times varied from 7.38 to 7.72 ms.

**Table 3:** Shock parameters for the single shock impact with helium driven shock.

Sequence	Gas	$P_4/P_1$ (MPa/MPa)	$P_5$ (MPa)	$t_d$ (ms)	Mach number ($M_s$)	$I^{+}$ (Pa s)	$p_{avg,1}$ (kPa)
SRSP-I	Helium/air	4.31/0.07	0.38	7.84	2.89	186.4	23.8
SRSP-II	Helium/air	4.35/0.07	0.39	7.81	2.95	200.9	25.6
SRSP-III	Helium/air	4.27/0.07	0.37	7.85	2.90	201.7	25.7
SRSP-IV	Helium/air	4.36/0.07	0.38	7.86	2.90	181.5	23.1
SRSP-V	Helium/air	4.23/0.07	0.36	7.85	2.91	208.4	26.6

**Table 4:** Shock parameters for the second shock impact with helium driven shock.

Sequence	Gas	$P_4/P_1$ (MPa/MPa)	$P_5$ (MPa)	$t_d$ (ms)	Mach number ($M_s$)	$I^{+}$ (Pa s)	$p_{avg,2}$ (kPa)
SRSP-I	Helium/air	4.54/0.07	0.43	7.44	3.25	209.8	26.7
SRSP-II	Helium/air	4.65/0.07	0.46	7.72	3.34	220.2	28.1
SRSP-III	Helium/air	4.28/0.07	0.39	7.61	3.12	217.3	27.7
SRSP-IV	Helium/air	4.46/0.07	0.42	7.42	3.21	193.1	24.6
SRSP-V	Helium/air	4.55/0.07	0.45	7.38	3.22	216.6	27.6

In Fig. 12a,b respectively, the backplate deformation for the sequences after single and double shock impact have been shown. As the final line of defence for the armour, the backplate must exhibit the least amount of deformation to protect the body or structure from impact. The greater the dimple, the lower the capability of the material to shield^33^.The least backplate deformation ($\sim$ 0.45 mm) after the single shock hit was observed in the SRSP-II, followed by SRSP-IV, and SRSP-III. SRSP-V exhibited significantly greater backplate deformation ($\sim$ 2.45 mm), followed by SRSP-I ($\sim$ 0.9 mm). As demonstrated in the compositions SRSP-II, SRSP-IV, and SRSP-III, the presence of paperboard seemed to have reduced the backplate deflection. The shock impedance mismatch induced by appropriate positioning of the paperboard layer next to the AA6061-T6 backplate, has contributed to the shock wave dissipation through the wave phenomena of reflection and transmission^47^. After the second shock hit, the backplate deformation was aggravated. The least damaged sequences among the others were SRSP-IV and SRSP-II (deformation $\sim$ 1.3–1.45 mm). However, the increase in deformation after the second shock hit for SRSP-III was approximately 196%, whereas the increase was $\sim$ 232% for SRSP-II and SRSP-IV. This shows that the residual plastic deformation in the layers during the first shock hit, aggravate further during the successive shock hit. This trend in the backplate deformation was also observed in Ref.^48^.

Figure 13a,b illustrates the faceplate deformation for the sequences after single shock and double shock impact. The deformation readings were interpreted as negative since the faceplate and backplate deform in the same direction, the deformation is detrimental to the structure being protected. Although the degree of deformation does not necessarily indicate that the material safeguarding the structure has been damaged, further shock waves impinging on the layers along with any shrapnel could breach the face plate and penetrate the core and beyond. The SRSP-II, SRSP-IV, and SRSP-V sequences showed the lowest deflections. The paperboard often makes up the penultimate layer of these three layers and appears to contribute to the decreased faceplate deflection. A much larger value of − 0.93 mm was displayed by SRSP-I, followed by a somewhat higher result ($\sim$ − 0.55 mm) in SRSP-III, where the paperboard is the third layer. The deformation in SRSP-IV and SRSP-II ranged from − 0.38 to − 0.46 mm. For the SRSP-I, SRSP-II, SRSP-III, and SRSP-IV, the faceplate deformation increased significantly ($\sim$ 2–2.7 times) after the second shock, whereas the SRSP-V showed the least face layer deformation. As mentioned earlier, the residual plastic deformation in the plies worsen the deformation during the second shock hit, due to the plasticity effects. The SRSP-II and SRSP-IV showed the least backplate and faceplate deformations based on the twin shock impact studies. Similar response was observed in Ref.^48^, although the deformation profile was comparatively smaller.

### Results of the numerical model for shock impact on the FMLs

Figure 12c,d respectively show the deformation profiles for the various sequences after single and double shock impact, as computed from the numerical model. It is to be noted that the numerical model gives the deformation profile for the entire laminate which was taken up for comparison with the backplate deformation obtained from the experiments. Comparing Fig. 12a,c, SRSP-I, SRSP-II, SRSP-III, and SRSP-IV showed a good agreement for the backplate deformation after single shock hit between experimental and numerical results. But, in case of SRSP-V, the maximum backplate deformation from experiment was − 2.5 mm after single shock hit, while the numerical model showed a value of − 1.4 mm. Similarly, on comparing Fig. 12b,d, after the double shock hit, only SRSP-I showed good agreement between the experimental and numerical maximum backplate deformations. But, for the sequences SRSP-II, SRSP-III, SRSP-IV, and SRSP-V, the numerical model was underpredicting the maximum backplate deformation by 48–67%. Hence, the numerical model underpredicts the backplate deformation by a large extent for the laminates.

Figure13c,d show the numerical results for the deformations of the various sequences which is same as that shown in Fig. 12c,d, taken up here for comparison with the faceplate deformation obtained from the shock impact experiments from helium-driven shocks. From Fig. 13a,c, the deformation values predicted by the numerical model for single shock impact, were in close agreement with the faceplate deformations values for the sequences SRSP-I, SRSP-II, SRSP-III and SRSP-IV. However, for the sequences SRSP-V, the numerical model overpredicted the faceplate deformation by a factor of 5.2. Likewise, on comparing Fig. 13b,d, the deformations computed from the numerical model for the second shock impact were in moderate agreement to the faceplate deformations of SRSP-I, SRSP-II, SRSP-III and SRSP-IV after the second shock impact. The variations of 16–32% were observed for the sequences. The numerical model severely overpredicted the faceplate deformation of SRSP-V, similar to what it had done in the case of the single shock hit. The large difference between numerical values of deformation and the faceplate deformation can be attributed to the idealistic assumptions using the CLT, where the ply interfaces were considered as perfectly bonded without considering the shockwave interactions at the interfaces^15,40^. Also, the numerical model lacks the deformation term associated with the fluid-structure interaction as the shock wave collides with the laminate.

**Table 5:** Maximum transverse deformation of the sequences for helium-driven shock impact.

Sequence	$w_{max,1}$ (mm)	$\frac{w_{max,1}}{t} (\%)$	$w_{max,2}$ (mm)	$\frac{w_{max,2}}{t} (\%)$
SRSP-I	$-$ 0.98	$-$ 39.03	$-$ 1.82	$-$ 82.96
SRSP-II	$-$ 0.37	$-$ 10.17	$-$ 0.64	$-$ 21.33
SRSP-III	$-$ 0.40	$-$ 11.51	$-$ 0.71	$-$ 23.90
SRSP-IV	$-$ 0.36	$-$ 10.33	$-$ 0.65	$-$ 21.34
SRSP-V	$-$ 1.26	$-$ 44.95	$-$ 2.08	$-$ 91.64

### Validation of the experimental results

#### Dimensionless cumulative deformation for the sequences

15 $$\begin{aligned} \Delta _c= & {} \frac{\delta _{b}-\delta _{f}}{t} \end{aligned}$$

16 $$\begin{aligned} \Delta _{c_{num}}=\frac{2\delta _{num}}{t}=\frac{2w_{2}}{t} \end{aligned}$$

**Figure 14:**
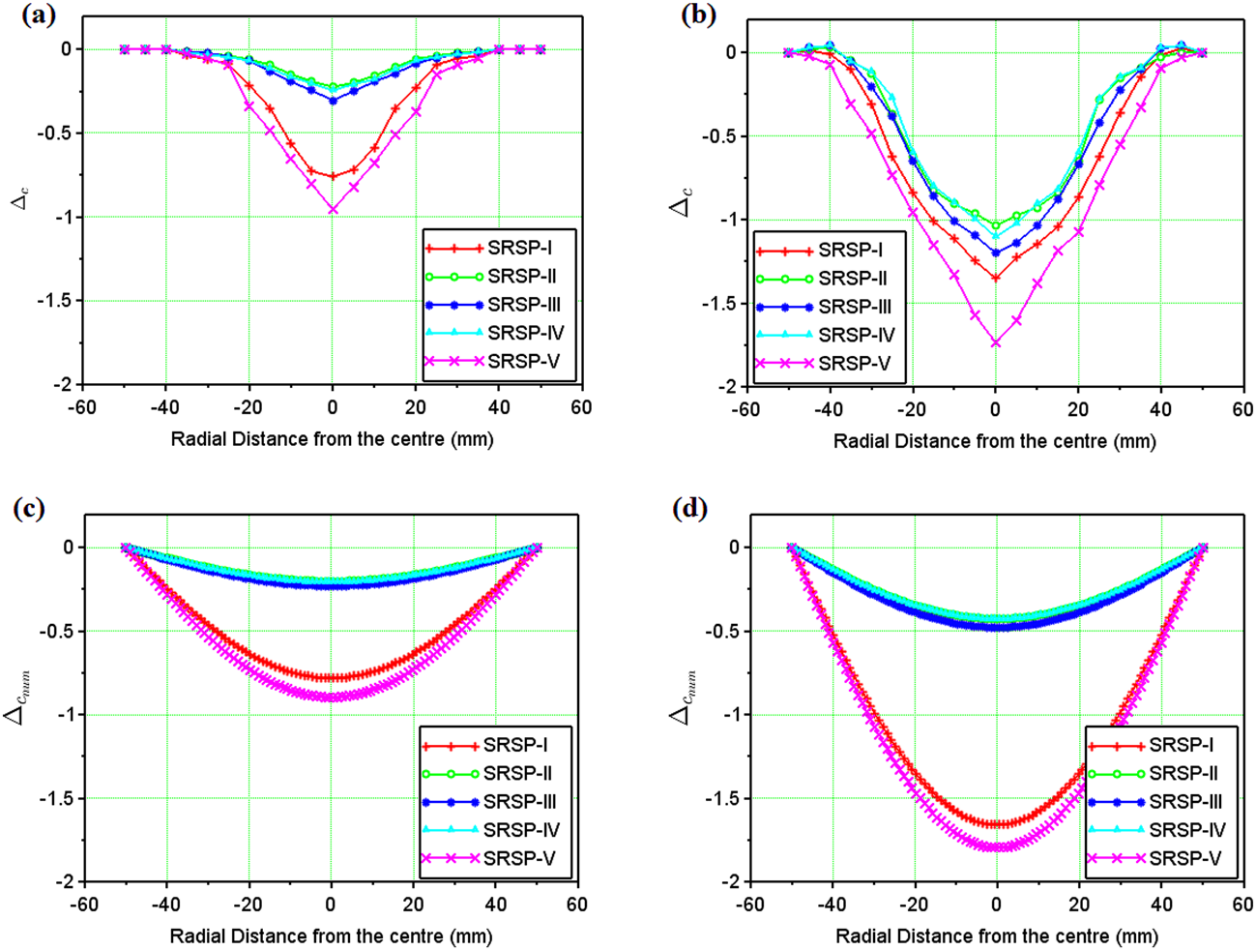
Dimensionless cumulative deformations $\Delta _c$ and $\Delta _{c_{num}}$ of the laminates (**a**) Single Hit (experimental) (**b**) Double Hit (experimental) (**c**) Single Hit (Numerical) (**d**) Double Hit (Numerical).

**Figure 15:**
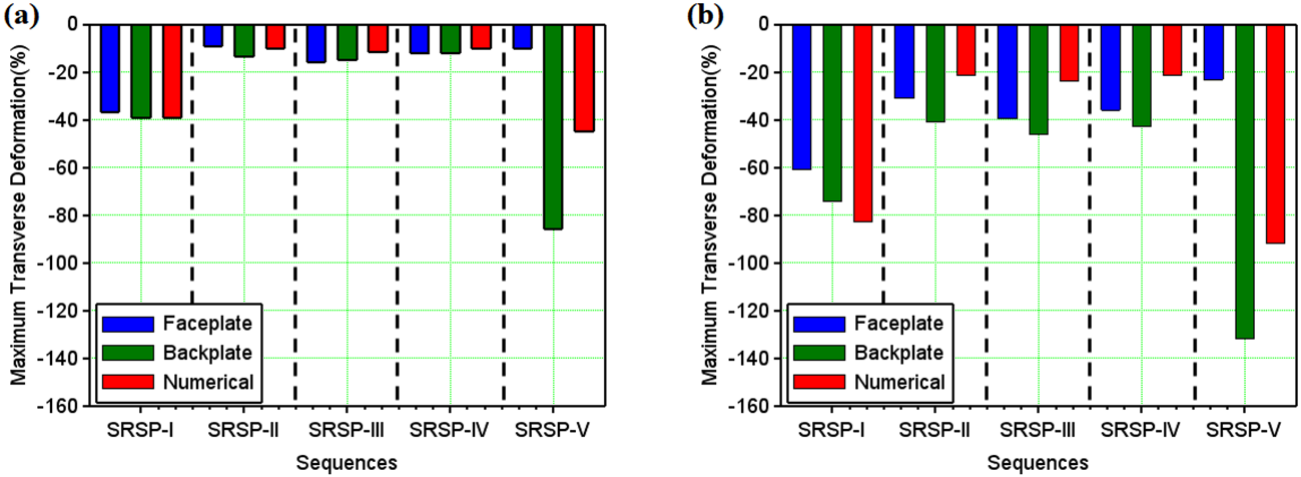
Maximum transverse deformation (%) of the laminates (**a**) Single Hit (**b**) Double Hit.

The face and the back layers distort in the direction of the shock impact, hence, a dimensionless cumulative deformation ($\Delta _c$) was defined as given in Eq. (15), which is the ratio of the algebraic difference between the backplate deformation ($\delta _{b}$) and the faceplate deformation ($\delta _{f}$) for a sequence, to the relevant laminate thickness (*t*). For the results of the numerical model, the deformation was predicted for the entire laminate without differentiating the faceplate and backplate deformations. Hence, the relative deformation given in Eq. (16), was compared with the dimensionless cumulative deformation in Fig. 14. These results demonstrate the significance of the AA6061 faceplate in reducing the shock impact, even though SRSP-V showed the highest % deformation for the backplate and the second largest percentage deformation for the faceplate. The least amount of percentage deformation was seen in SRSP-II, then SRSP-IV, for both the faceplate and the backplate. The series SRSP-I showed the largest percentage deformation of the faceplate, emphasising the value of paperboard as an intermediary layer. Of all sequences, SRSP-II and SRSP-IV have the least overall deformation. The second-highest overall deformation is shown by SRSP-I, demonstrating that the absence of the paperboard ply diminishes the ability of the laminate to absorb the shock energy. The paperboard layer between the aramid-epoxy and UHMWPE-epoxy core laminae maintains the benefit of the low shock impedance layer, displaying a deformation which is $\sim$ 11% greater than that of SRSP-II and SRSP-IV. The influence of the paperboard-epoxy ply location has been studied in Ref.^48^ and is in agreement with the current findings. The maximum deformation values computed by the numerical model for the helium-driven shock impact on the sequences have been tabulated in Table 5. For helium driver gas, the numerical model was found to underpredict the maximum deformation values of the backplate as seen in Fig. 15. This can be attributed to the fact that the model was based on the CLT as defined before, which disregards the plasticity effects in the individual plies^44–46^. However, the maximum deformation for the sequences SRSP-I, SRSP-II, SRSP-III, and SRSP-IV could be predicted very accurately by the numerical model. The numerical result for SRSP-V, however, was rather close to the backplate deformation (experimental), while substantial difference between the faceplate deformation values between experimental and numerical results was observed. Also, the discrepancies in the spatial deformation values for the sequences can be attributed to the formation and propagation of the shock wave inside the circular section at the juncture of diaphragm fracture. The shock velocity profiles resulted in lower deformation for the lower sectional radii, and at the centre of the shock tube, maximum shock velocity was responsible for high central deformation across sequences.

### Comparison of nitrogen-driven and helium-driven shock impact on the FMLs

**Figure 16:**
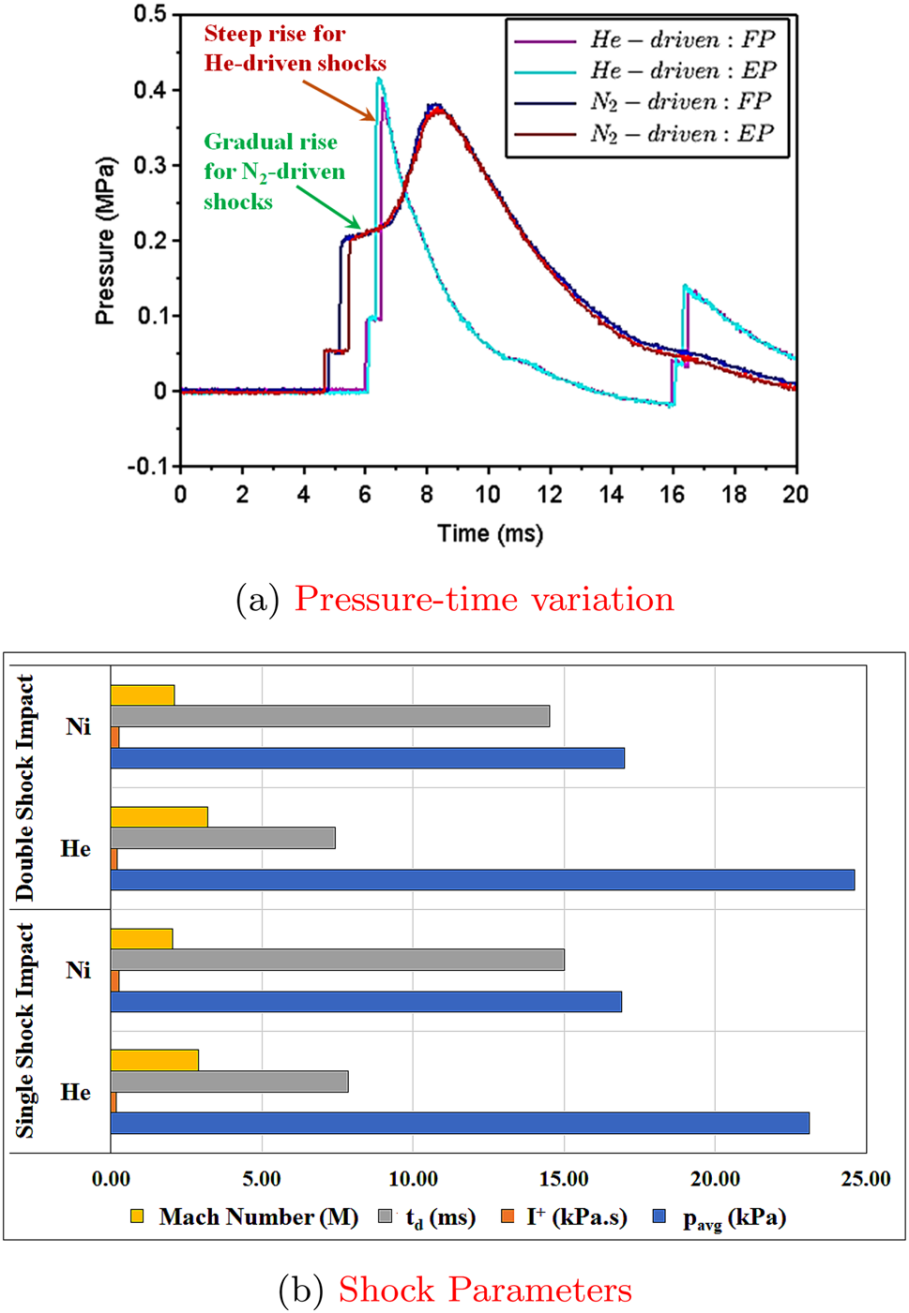
Comparison of Helium-driven and Nitrogen-driven shock waves^48^ for SRSP-IV.

The nitrogen-driven shock impact studies on the FMLs were carried out in our earlier work^48^. The results obtained from the N$_2$ driven shock impact studies were utilized for comparison with helium-driven shock wave characterization. The pressure-time histories and shock parameters for the helium-driven and nitrogen-driven shock waves are shown in Fig. 16. The rise in the pressures (detected by FP and EP sensors) for helium driven shock was steeper compared to the gradual increase in pressures for the nitrogen driven shock for the same FML sequence. The decay rate for helium driven shock and nitrogen driven shock are seen in Fig. 16a.

**Figure 17:**
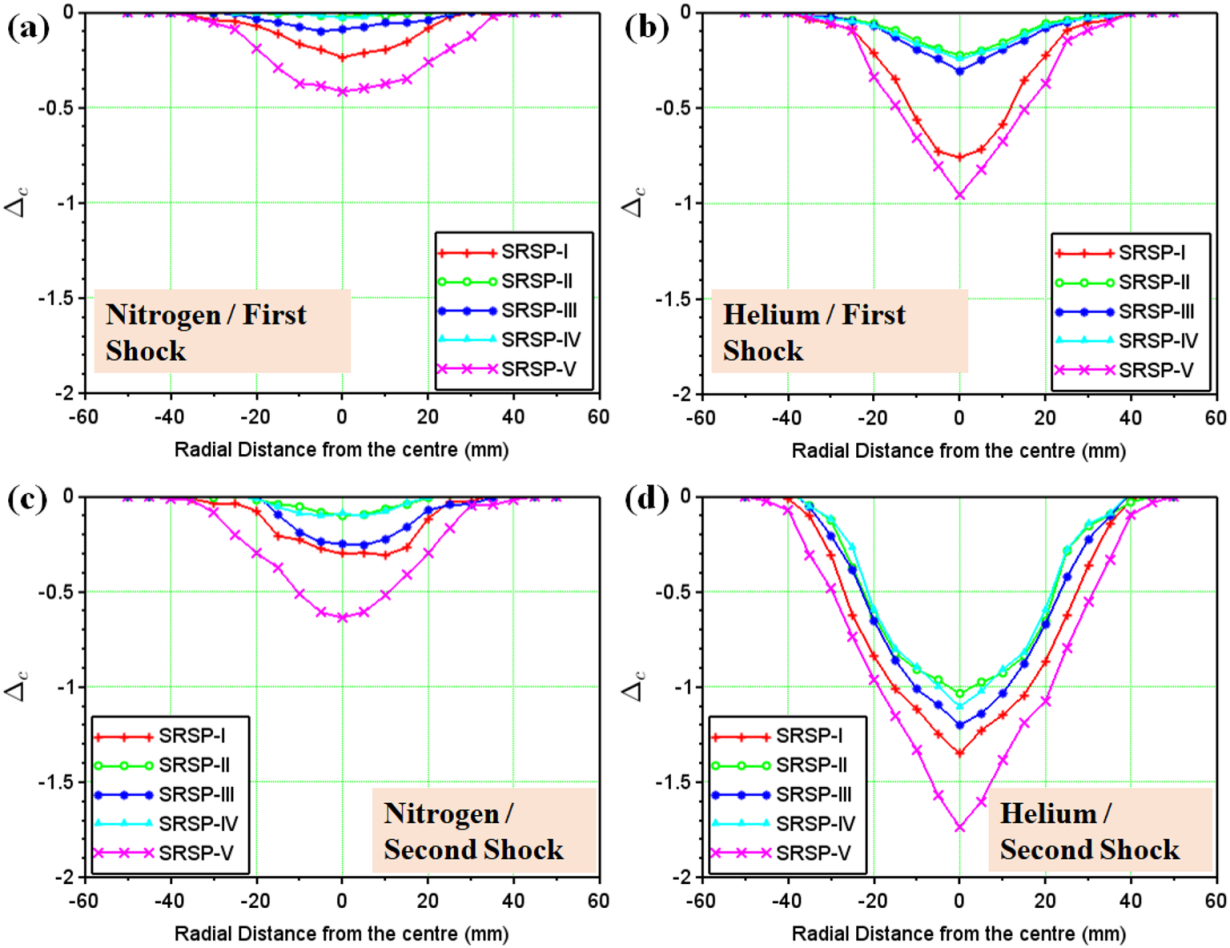
Dimensionless cumulative deformation $\Delta _c$ of the laminates (**a**) Nitrogen driven shock (first) (**b**) Helium driven shock (first) (**c**) Nitrogen driven shock (second) (**d**) Helium driven shock (second).

From Fig. 16b, in case of diaphragms with same thickness and material, helium driven shocks showed a higher average pressure, higher Mach numbers, and lower decay times for the first and second shock impact as compared to the nitrogen driven shocks.The low decay time in helium-driven shocks was half the decay time observed in nitrogen-driven shock waves. Helium is an inert gas while Nitrogen is a di-atomic gas. Also, the mass diffusivity of helium is the second highest (after hydrogen). Inside the shock tube, when di-atomic gases are used as a driver, they result in weaker shock waves, and suffer changes in the molecular rotational and vibration energies, which necessitate many inter-molecular collisions to achieve equilibrium with the translational motion of the shock^5,31,32,34^. Hence, nitrogen driven shocks showed higher decay times, lower average pressures, lower shock velocities, and in turn lower Mach Numbers as compared to helium driver gas. Figure 17 compares the cumulative dimensionless deformation of the laminates (entire laminate) after first and second shock wave impact for nitrogen and helium as the driver gases respectively. On first wave impact by nitrogen driven shock, SRSP-V showed the highest value of $\Delta _c$ (= − 0.4), followed by SRSP-I ($\Delta _c$ = − 0.2). After the second wave impact by nitrogen driven shock, SRSP-V showed an increase in $\Delta _c$ value to − 0.7, while that of SRSP-I increased to − 0.3. When helium was used as a driver gas, on first wave impact on the laminates, the value of $\Delta _c$ for SRSP-V was − 0.95, while that for SRSP-I was − 0.7. After second wave impact by helium driven shock, there was a staggering rise in the values of $\Delta _c$ in case of all the laminates. The value of $\Delta _c$ for SRSP-V soared to − 1.8, while that for SRSP-I rose to − 1.3. For the laminates SRSP-IV, SRSP-II, and SRSP-III, the values of $\Delta _c$ ($\sim$ 0.9–1.1) was observed. Evidently, helium driven shocks induced significant damage to the laminates as compared to nitrogen driven shocks.

### CBC tomography image analysis

**Figure 18:**
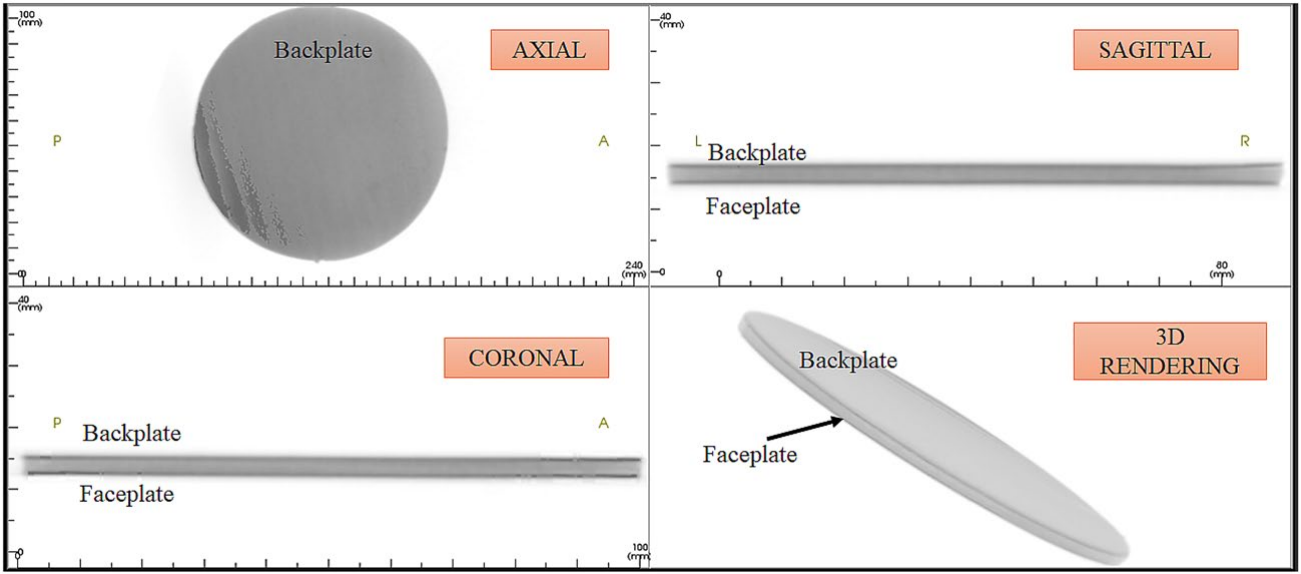
CBCT images of SRSP-I as-machined specimen (before shock impact).

**Figure 19:**
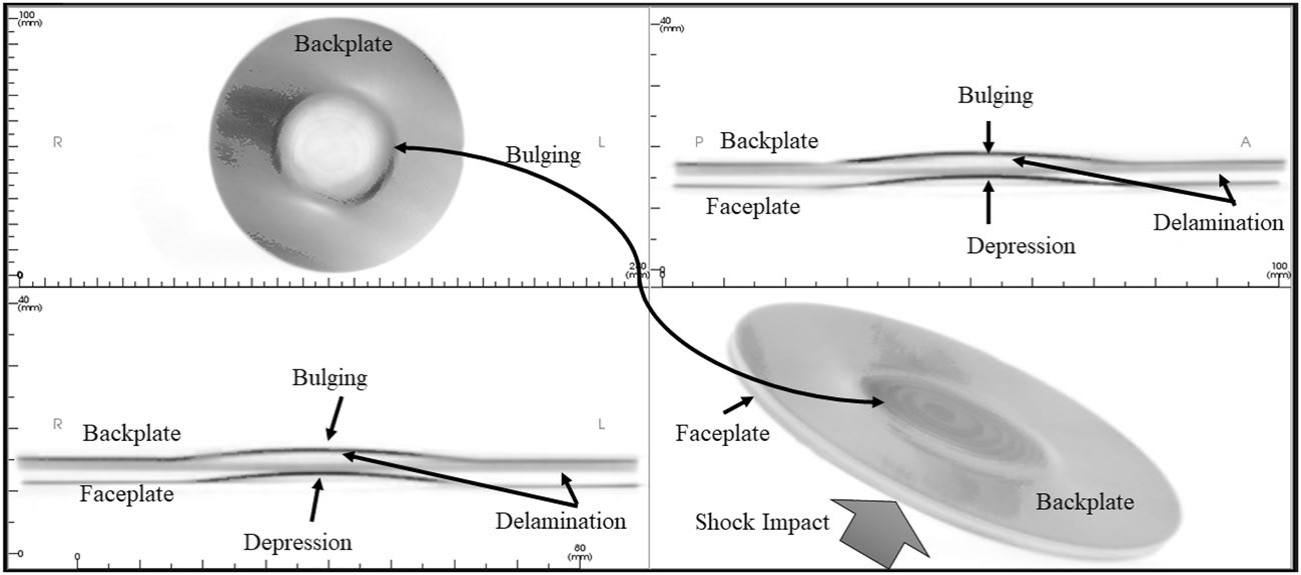
CBCT images of SRSP-I subjected to the helium-driven shock impact.

**Figure 20:**
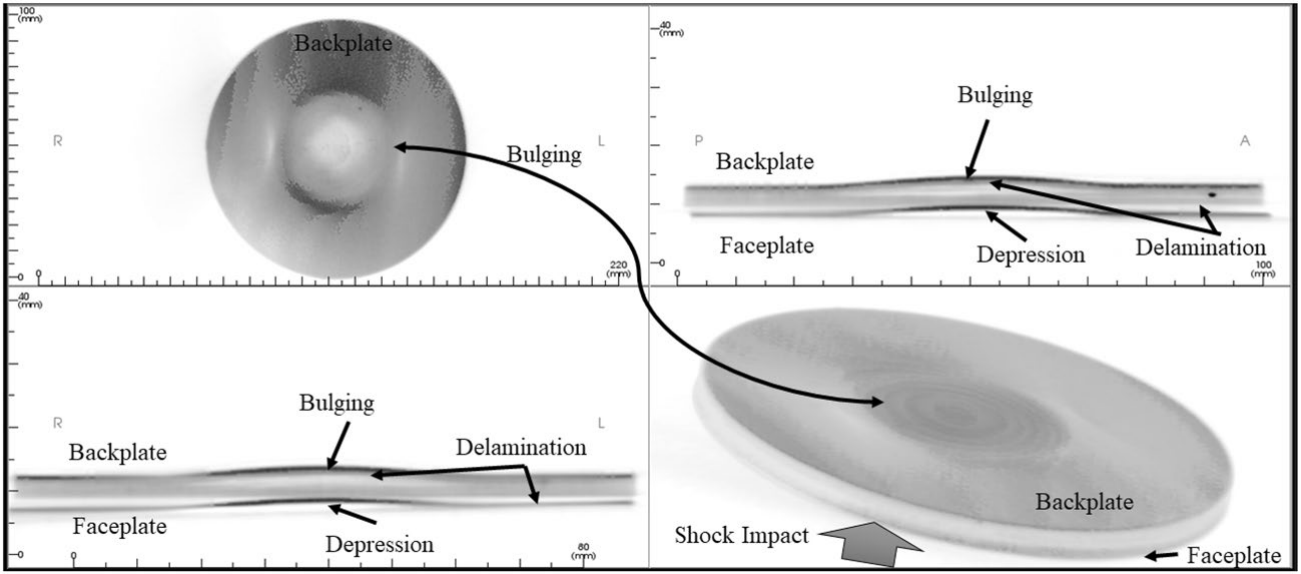
CBCT images of SRSP-II subjected to the helium-driven shock impact.

**Figure 21:**
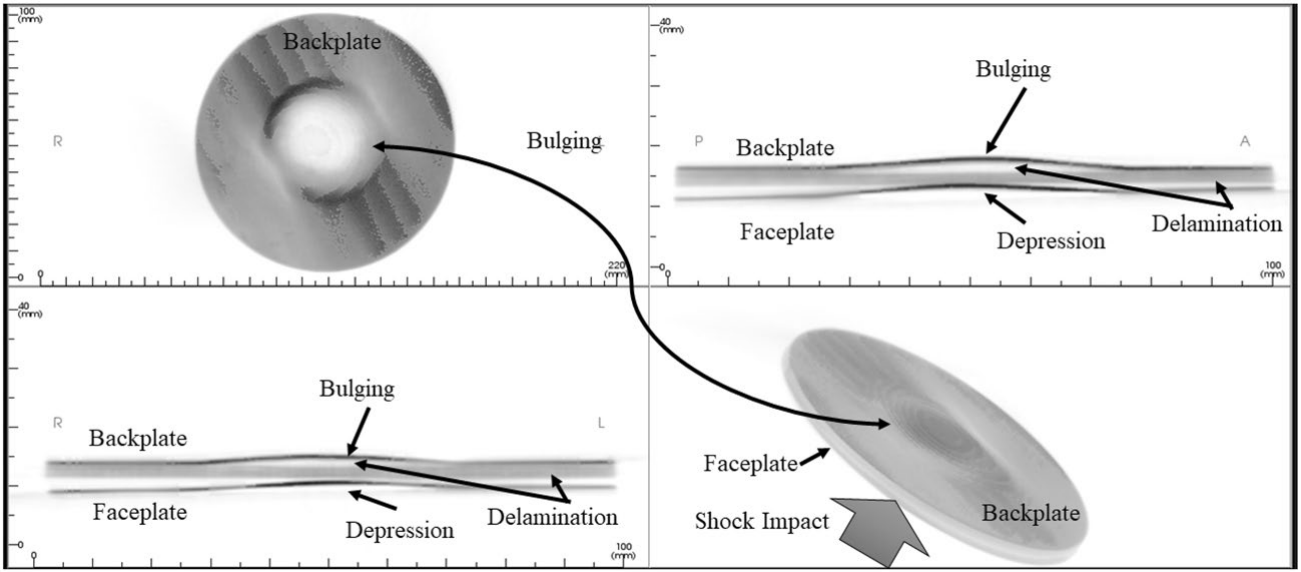
CBCT images of SRSP-III subjected to the helium-driven shock impact.

**Figure 22:**
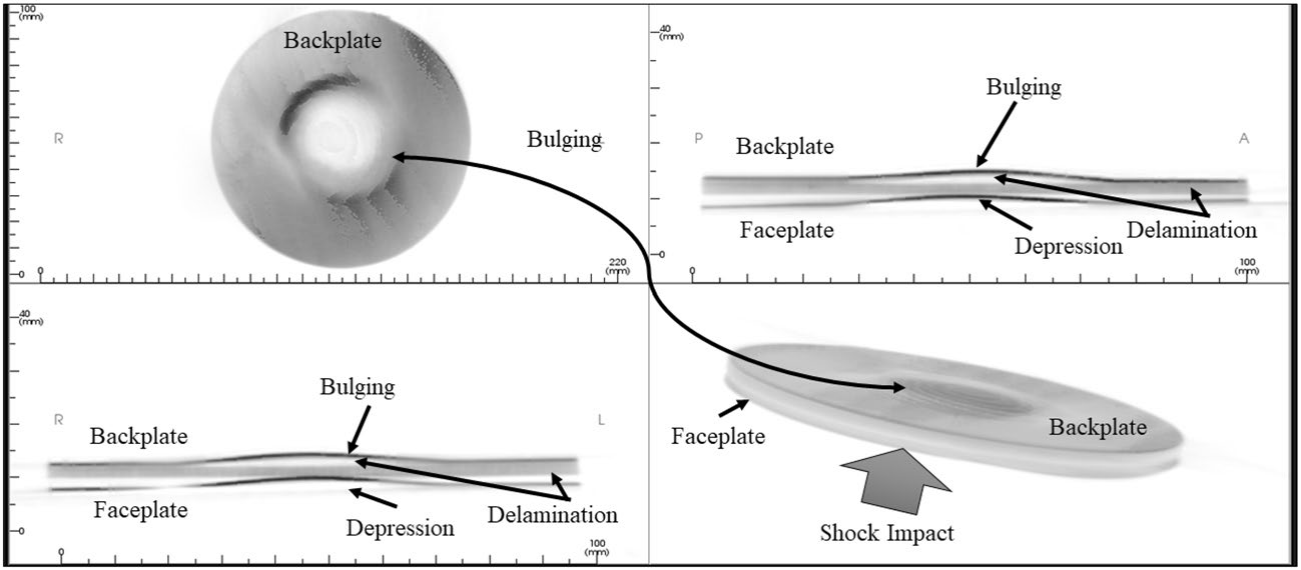
CBCT images of SRSP-IV subjected to the helium-driven shock impact.

**Figure 23:**
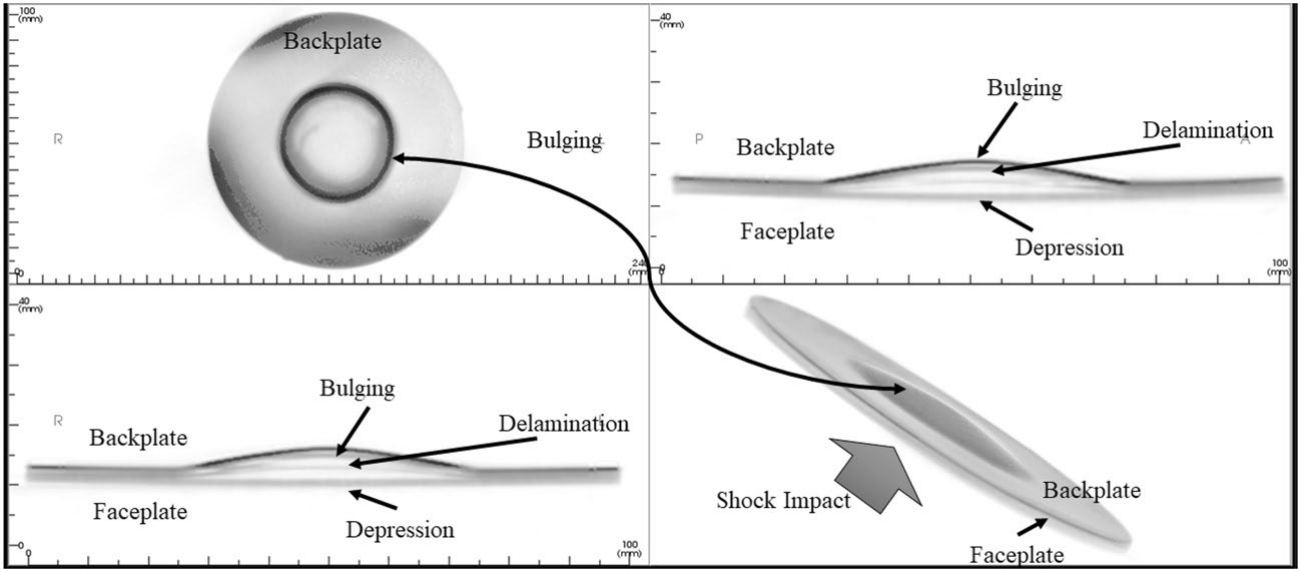
CBCT images of SRSP-V subjected to the helium-driven shock impact.

Figure 18 shows the as-machined specimen of SRSP-I sequence before being subjected to the shock impact. There are four views available for inspection. The “axial” view shows the sequence top view (with backplate in focus), “sagittal” gives the side view, “coronal: gives the front view, while the 3D rendering is shown in the bottom right box of Fig. 18. When the shock tube experiments were conducted on the sequences, the post impact specimens were again subjected to CBCT scanning The CBCT scanned images for the various sequences are shown in Fig. 19 for SRSP-I (post impact), Fig. 20 for SRSP-II (post impact), Fig. 21 for SRSP-III (post impact), Fig. 22 for SRSP-IV (post impact), Fig. 23 for SRSP-V (post impact) respectively.

From the deformation patterns obtained by the sagittal/coronal views, it was seen that SRSP-II, SRSP-IV, and SRSP-III had least backplate deformation, which was consistent with the deflection values determined from the dial gauge readings. Across the sequences, severe delamination was observed central region of the backplate and at the outer periphery of the faceplate for the sequences SRSP-I, SRSP-II. SRSP-III and SRSP-IV. In case of the SRSP-V, the faceplate was aramid-epoxy play which showed minimal plastic deformation (no delamination at the faceplate-UHMWPE interface seen), while the AA6061-T6 backplate protruded significantly due to the shock impact as seen in Fig. 23. The depressions on the faceplate and the protrusion on the backplate from the CBCT scanning were symmetric at the centre of the circular plates.

## Conclusions

The primary objective of investigating the response of different stackup sequences to repeated helium-driven shock impacts was successfully achieved. The experimental results, encompassing actual deformation, maximum deformation, and dimensionless cumulative deformation, were examined. These findings were compared against the outcomes generated by numerical simulations. By juxtaposing the deformation profiles of various stackups subjected to helium-driven twin shock impacts with those from nitrogen-driven twin shock impacts, noteworthy distinctions were observed. Beyond the evident distinctions in higher Mach numbers and quicker decay times associated with helium-driven shocks, it became evident that such shocks intensified depression, bulging, and delamination failures within the laminates. The shock-impacted specimens were subjected to CBCT scanning and the modes of failure in each of the laminates were scrutinized. The following conclusions were drawn :

- Mach numbers ($\sim$ 2.9–3.3) were achieved using helium as the driving gas (with $\gamma$ = 1.67), the impact effects of which are comparable to those exerted by high intensity blast waves in the nearfield of detonation sites (M $\sim$ 3.5–4.0).
- The decreased deformations displayed by SRSP-II, SRSP-IV, and SRSP-III in comparison to SRSP-I, indicate that using paperboard-epoxy ply as a central layer to induce shock impedance mismatch can positively influence the FML response.
- The maximum backplate deformation among the sequences, observed in SRSP-V was caused by the absence of the AA6061-T6 faceplate. As a result, the presence of a metallic face layer of high shock impedance is essential for FMLs for effective shock energy attenuation.
- The numerical model yielded moderately accurate results for faceplate deformation in SRSP-I, SRSP-II, SRSP-III, and SRSP-IV sequences. However, a notable disparity was observed in SRSP-V between the numerical and experimental deformation. Moreover, the numerical model consistently underestimated backplate deformations across all sequences.
- There was an excellent agreement between the cumulative dimensionless deformation from shocktube experiments and the relative deformation predicted by the numerical model across all sequences, which proves that the numerical model can be successfully utilized to capture the shockwave impact responses for laminates.
- As for the shock impact effect on the materials of the FMLs, successive impacts cause the deformation of the FMLs to increase drastically, indicating that thin-FMLs can sustain up to a maximum of twin shock impacts.
- Helium-driven shocks severely deformed the specimens, with the second shock impact leading to the delamination of AA6061 plates, as inferred from the CBCT scanned images of the specimens. The backplates showed core delamination while the faceplates showed delamination at the peripheral annulus region.

Among all the sequences, SRSP-II and SRSP-IV emerged as ideal laminate sequences for use as effective shielding configurations against multiple shock impacts, as they showed the least backplate deformation essential to safeguard any structure lying beyond the shield. The functional grading of the sequences based on shock impedance matching could be advantageous for shock impact resistance, and could be extended to other material systems in multi-layered sandwich structures.

## Data Availability

The datasets used and/or analysed during the current study are available from the corresponding author on reasonable request.

## List of symbols

$\Delta _c$: Dimensionless cumulative deformation of laminate
$\Delta _{c_{num}}$: Relative deformation of laminate
$\delta _{f}$: Deformation of backplate (mm)
$\delta _{f}$: Deformation of faceplate (mm)
$\delta _{num}$: Numerical deformation of laminate (mm) which is equal to the transverse deflection w$_1$ for the sequence
$\kappa$: Curvature of the beam (/m)
$\left( E_{x}\right) _{j}$: Longitudinal Modulus of *j* *th* ply along global *x*-direction (GPa)
*A*: Flow area (m$$^2$$)
*b*: Width of the beam (mm) (directed normal to the viewing plane)
*D*: Diameter of the specimen (= 100 mm)
$$I^+$$: Positive impulse
$$I^-$$: Negative impulse
$M_b$: Applied bending moment (Nm)
*P*: Wave pressure (kPa)
$P(\tau )$: Wave pressure at time instant ‘*t*’ (kPa)
$p_{avg,1}$: Average pressure in kPa for single shock impact
$p_{avg,2}$: Average pressure in kPa for second shock impact
*r*: Radius from the center of the plate (mm) (0 $\le$ *r* $\le$ $\frac{D}{2}$)
*t*: Laminate thickness (mm)
$w_1$: Deflection at a given radius ‘*r*’ in mm for single shock impact
$w_2$: Deflection at a given radius ‘*r*’ in mm for second shock impact
$w_{max,1}$: Maximum transverse deflection of the plate during single shock impact (mm)
$w_{max,2}$: Maximum transverse deflection of the plate after second shock impact(mm)
$z_{j}$: Distance from the geometric mid-plane to the outside of *j* *th* ply
$P_0$: Ambient pressure at ‘*t*’=0 (kPa)
${\Delta }(mu)$: Rate of change in momentum (kJ/s)
